# Non‐invasive diagnosis and surveillance of bladder cancer with driver and passenger DNA methylation in a prospective cohort study

**DOI:** 10.1002/ctm2.1008

**Published:** 2022-08-15

**Authors:** Yu Xiao, Lingao Ju, Kaiyu Qian, Wan Jin, Gang Wang, Yan Zhao, Wei Jiang, Nan Liu, Kai Wu, Minsheng Peng, Rui Cao, Sheng Li, Hongjie Shi, Yan Gong, Hang Zheng, Tongzu Liu, Yongwen Luo, Haoli Ma, Luyuan Chang, Gang Li, Xinyue Cao, Ye Tian, Zilin Xu, Zhonghua Yang, Liuying Shan, Zhongqiang Guo, Dongai Yao, Xianlong Zhou, Xintong Chen, Zicheng Guo, Dongmei Liu, Song Xu, Chundong Ji, Fang Yu, Xin Hong, Jun Luo, Hong Cao, Yi Zhang, Xinghuan Wang

**Affiliations:** ^1^ Department of Urology Zhongnan Hospital of Wuhan University Wuhan China; ^2^ Department of Biological Repositories Zhongnan Hospital of Wuhan University Wuhan China; ^3^ Human Genetic Resource Preservation Center of Hubei Province Wuhan China; ^4^ Euler Technology ZGC Life Science Park Beijing China; ^5^ Wuhan Research Center for Infectious Diseases and Cancer Chinese Academy of Medical Sciences Wuhan China; ^6^ Emergency Center Zhongnan Hospital of Wuhan University Wuhan China; ^7^ Hubei Clinical Research Center for Emergency and Resuscitation Zhongnan Hospital of Wuhan University Wuhan China; ^8^ Medical Research Institute Wuhan University Wuhan China; ^9^ State Key Laboratory of Genetic Resources and Evolution, Kunming Institute of Zoology Chinese Academy of Sciences Kunming China; ^10^ University of Academy of Sciences Kunming College of Life Sciences Kunming China; ^11^ Department of Urology, Beijing Friendship Hospital Capital Medical University Beijing China; ^12^ Cancer Precision Diagnosis and Treatment and Translational Medicine Hubei Engineering Research Center Zhongnan Hospital of Wuhan University Wuhan China; ^13^ Clinical Trial Center Zhongnan Hospital of Wuhan University Wuhan China; ^14^ Physical Examination Center Zhongnan Hospital of Wuhan University Wuhan China; ^15^ Department of Urology The Central Hospital of Enshi Tujia and Miao Autonomous Prefecture Enshi China; ^16^ Department of Urology The Affiliated Hospital of Panzhihua University Panzhihua China; ^17^ Department of Pathology Zhongnan Hospital of Wuhan University Wuhan China; ^18^ Department of Urology Peking University International Hospital Beijing China

**Keywords:** bladder cancer, diagnosis and prognosis, methylation, non‐invasive screening, prospective cohort study, urine tumour DNA

## Abstract

**Background:**

State‐of‐art non‐invasive diagnosis processes for bladder cancer (BLCA) harbour shortcomings such as low sensitivity and specificity, unable to distinguish between high‐ (HG) and low‐grade (LG) tumours, as well as inability to differentiate muscle‐invasive bladder cancer (MIBC) and non‐muscle‐invasive bladder cancer (NMIBC). This study investigates a comprehensive characterization of the entire DNA methylation (DNAm) landscape of BLCA to determine the relevant biomarkers for the non‐invasive diagnosis of BLCA.

**Methods:**

A total of 304 samples from 224 donors were enrolled in this multi‐centre, prospective cohort study. BLCA‐specific DNAm signature discovery was carried out with genome‐wide bisulfite sequencing in 32 tumour tissues and 12 normal urine samples. A targeted sequencing assay for BLCA‐specific DNAm signatures was developed to categorize tumour tissue against normal urine, or MIBC against NMIBC. Independent validation was performed with targeted sequencing of 259 urine samples in a double‐blinded manner to determine the clinical diagnosis and prognosis value of DNAm‐based classification models. Functions of genomic region harbouring BLCA‐specific DNAm signature were validated with biological assays. Concordances of pathology to urine tumour DNA (circulating tumour DNA [ctDNA]) methylation, genomic mutations or other state‐of‐the‐art diagnosis methods were measured.

**Results:**

Genome‐wide DNAm profile could accurately classify LG tumour from HG tumour (LG NMIBC vs. HG NMIBC: *p* = .038; LG NMIBC vs. HG MIBC, *p* = .00032; HG NMIBC vs. HG MIBC: *p* = .82; Student's *t*‐test). Overall, the DNAm profile distinguishes MIBC from NMIBC and normal urine. Targeted‐sequencing‐based DNAm signature classifiers accurately classify LG NMIBC tissues from HG MIBC and could detect tumours in urine at a limit of detection of less than .5%. In tumour tissues, DNAm accurately classifies pathology, thus outperforming genomic mutation or RNA expression profiles. In the independent validation cohort, pre‐surgery urine ctDNA methylation outperforms fluorescence in situ hybridization (FISH) assay to detect HG BLCA (*n* = 54) with 100% sensitivity (95% CI: 82.5%–100%) and LG BLCA (*n* = 26) with 62% sensitivity (95% CI: 51.3%–72.7%), both at 100% specificity (non‐BLCA: *n* = 72; 95% CI: 84.1%–100%). Pre‐surgery urine ctDNA methylation signature correlates with pathology and predicts recurrence and metastasis. Post‐surgery urine ctDNA methylation (*n* = 61) accurately predicts recurrence‐free survival within 180 days, with 100% accuracy.

**Conclusion:**

With the discovery of BLCA‐specific DNAm signatures, targeted sequencing of ctDNA methylation outperforms FISH and DNA mutation to detect tumours, predict recurrence and make prognoses.

## Graphical abstract

**Graphical Headlights**:

(1) Development of statistical methods to determine bladder cancer (BLCA)‐specific DNA methylation (DNAm) profile for an accurate classification of high‐ (HG) and low‐grade (LG) BLCA.
(2) Development of sequencing assay to detect BLCA‐specific DNAm in urine.
(3) Validation of urine DNAm as highly relevant biomarkers for non‐invasive diagnosis, prognosis and monitoring of BLCA in a prospective cohort.

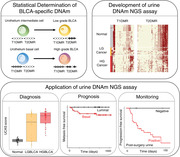

## 1 INTRODUCTION

A major challenge in managing urothelial carcinoma is the accurate diagnosis and classification of neoplastic lesions. The classification of bladder cancer (BLCA) describes two major subtypes, muscle‐invasive and non‐muscle‐invasive bladder cancer (MIBC/NMIBC), which differ significantly in treatment and management based on terms of their clinical manifestations and tumour biological behaviours. This makes accurate classification crucial before clinical decision‐making.^1^, ^2^, ^3^ The current classification standard for BLCA is based on a histopathological assessment of tissue biopsy or surgical resection specimens. Such a procedure is invasive, costly, and often associated with the risk of discomfort and infectious complications. Furthermore, assessment accuracy is complicated, with numerous shortcomings such as subjective evaluation by pathologists and the arbitrary selection of tissue samples.^4^, ^5^

Clinical treatment of BLCA depends on the accurate staging of tumour. Inaccurate staging results in reduced survival (in the case of understaging) or unnecessary surgery and permanent living quality loss (in the case of overstaging). The staging of BLCA relies on the tumour progress, that is, pathological manifest. In clinical practice, there are two classes of intermingled classification systems of urothelium cancer (>90% of BLCA), based on either tumour cell grading or muscle‐invasiveness. Pathologically, BLCA could be classified into MIBC or NMIBC, based on the presence of tumour cells invaded through lamina propria into the muscularis propria/detrusor layer of bladder. On the other hand, BLCA could be classified into low grade (LG) and high grade (HG), according to the morphology of tumour cells, the organization of tumour cells, the prevalence of cell division and the nucleus shape. Invasive tumours are classified as HG (traditionally G2/G3), and non‐invasive tumours are classified as LG.

Although LG tumours are never found muscle‐invasive according to the definition, HG tumours could be either muscle‐invasive or non‐muscle‐invasive, according to their stage of development. However, it is inappropriate to predict all LG tumours benign par the following reasons: (1) transformation (up‐grading) of LG tumours towards HG tumours is frequent and occurs in >30% of clinical cases; (2) manual classification of tumour grades might lead to clinical understaging of as many as 48% patients.^6^ Hence, accurate grading of BLCA is of pivotal importance.

Liquid biopsy with urine offers a promising alternative to tissue histopathology for diagnosis and monitoring of BLCA as it is easy to obtain and contains informative biomarkers, including cell‐free (cf) DNA, RNA and secreted proteins as well as dissociated tumour cells.^7^, ^8^ Existing non‐invasive, urine‐based diagnosis methods for BLCA target various aspects of cancer, such as aberrant methylation (methylation‐specific qPCR), DNA somatic mutation (circulating tumour DNA [ctDNA] sequencing), abnormal karyotype (FISH) or morphological changes (cytology analysis).^9^ Although widely applied in clinical practice, the previously mentioned methods harbour shortcomings such as low sensitivity, especially for detecting early, small, residual or recurrent tumours, as well as the inability to distinguish pathological subtypes.^10^, ^11^ The shortcomings of existing molecular diagnosis methods are attributable to limited specificity of the biomarkers, for example, distinguishing true tumour‐derived signals from a benign, non‐cancerous cell‐derived signal and limited sensitivity due to the low abundance of investigated subjects such as dissociated tumour cells in urine.

Aberrant DNA methylation (DNAm) is a pan‐cancer hallmark.^12^ Specific DNAm could be applied for cancer detection and a precise classification of cancer subtypes.^13^, ^14^, ^15^ An individual human body is generated from an identical genome as a multicellular ensemble of diverse cell types. During development, epigenetic modifications such as histone modification, differential histone subtype incorporation and DNA modification occur on the genome to selectively enhance or repress gene expression.^16^, ^17^, ^18^, ^19^, ^20^, ^21^ Such a process enables each cell to ‘read different books’ (express genes) from a similar ‘library’ (genome) to adopt different ‘majors’ (cell fate). Consequently, cells from various lineages show distinct genome‐wide DNAm profiles.^22^ Cancer‐associated DNAm could be resulted from two sources. First, cell‐type‐specific differential DNAm accompanies normal cell development and cell fate determination as a clonal outgrowth from a single mutated ancestor cell^23^; cancer inherited – partly – the epigenome from its ancestor. As a result, cell lineage‐associated DNAm could serve as a sensitive and specific biomarker to discriminate cancer cell‐of‐origin.^24^, ^25^, ^26^, ^27^ Second, widespread cancer‐specific DNAm changes occur during oncogenesis, creating a cancer‐specific DNAm profile that is drastically different from its normal counterparts.^28^, ^29^, ^30^, ^31^, ^32^ Although functionally largely unknown, such cancer‐specific DNAm has been widely applied as biomarkers to detect tumours.^25^, ^33^, ^34^, ^35^, ^36^, ^37^

Primarily, cancer‐associated DNAm is detected via a statistical comparison between DNAm profiles from groups of tissues, thus leading to the identification of differentially methylated region (DMR), genomic loci on which DNAm status is statistically different in one group of samples as compared to the other groups.^15^ Theoretically, cancer‐associated DMR could potentially result from four different scenarios: (1) inherited cell‐type‐specific DNAm signature from the clonal ancestor of cancer, which is a normal cell (Type I, T1DMR); (2) de novo DNAm shift during oncogenesis (Type II, T2DMR); (3) inherited cell‐type‐specific DNAm signature from a non‐cancer cell, which is not present in normal tissue (Type III, T3DMR), such as immune cells; or (4) de novo DNAm shift accompanied oncogenesis in a non‐cancer cell type (Type IV, T4DMR), such as reprogrammed fibroblast. However, in most prior work, these DMR subtypes were unclassified during the screening of tumour‐specific DNAm events.^38^, ^39^, ^40^ Consequently, the deduction of tumour‐derived fraction with DNAm on this DMR is limited by ‘contamination’ of the non‐tumour‐derived signal. Strategies to detect the ‘driver’, oncogenic (T2DMR) and ‘passenger’, tissue‐of‐origin‐specific (T1DMR) DNAm events in BLCA may significantly enhance specificity for DNAm‐based non‐invasive tumour detection.

Tumour‐derived DNAm is naturally relevant to tumour biology, less influenced by inter‐tumour heterogeneity and exists in large numbers compared to tumour‐specific DNA mutation. Assays using urine DNAm to detect or monitor BLCA have been reported in our previous study.^41^ A set of two methylated genes (TWIST1 and NID2) in urine samples have high (≥90%) sensitivity for the presence of primary BLCA.^39^ The UroMark assay (a 150 CpG loci biomarker panel) has a sensitivity of 98% for the detection of primary BLCA.^42^ Bladder EpiCheck, a urine test based on 15 methylation markers, displayed a sensitivity of 67% for NMIBC and 91.7% in MIBC recurrences with a specificity of 88% in a clinical trial.^43^ UtMeMa, another urine DNAm‐based mass spectrometry test, demonstrated 64.5%–100% sensitivity to Ta to T4 disease (overall 84.6%), with an overall specificity of 83.1%.^40^ Compared to state‐of‐the‐art clinical assays such as urine cytology or FISH and other assays focusing on detecting DNA mutation^44^, ^45^, ^46^ or copy number variation^45^, ^47^ in urine, DNAm‐based assay showed improved sensitivity but lower specificity. Additionally, most urine‐based DNAm assays were designed for and applied to diagnose BLCA, and none of them has been demonstrated to enable non‐invasive BLCA classification. Consequently, most of these tests have not been implemented in clinical practice.^4^

We reasoned that the shortcomings of urine‐based DNAm assays are caused by the limited specificity of the biomarkers inherited from their surrogate nature. Statistical comparison between arbitrarily selected groups of tissues may be confounded by cofactors unaware to the investigators. Furthermore, it is difficult to exclude all possible interference factors such as normal‐cell‐derived DNAm or DNAm introduced during intravesical chemotherapy (e.g. by gemcitabine).^48^ We depict that combining multi‐omics single‐cell analysis with statistical inference enables the accurate identification and classification of tumour‐derived DNAm events with superior specificity. The results revealed T2DMR DNAm as oncogenic events, which are strongly associated with tumour transformation, and T1DMR are inherited from the ancestral cell of neoplasm. Separating the two classes of DMR helped us identify oncogenic SOX2 enhancers modified by DNAm and further stratify LG BLCA by the prevalence of cancer‐specific T2DMR haplotypes. Methylation haplotypes on these DMRs are highly specific for tumours and indicate tumour class and grade.

**FIGURE 1:**
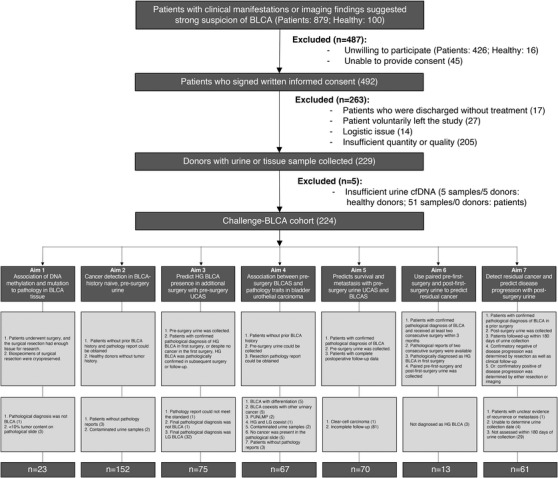
Flow chart of the study.

We have previously shown distinct DNAm alterations associated with BLCA subtypes originating from various lineages and tumour malignancy grades.^41^ This study aims to determine the source and nature of the previously mentioned differential DNAm. Furthermore, we tested whether estimating the ctDNA fraction from urine by sequencing cell‐free DNA (cfDNA) methylation on these precisely defined tumour‐derived DMR could be used to non‐invasively detect and classify BLCA in a multi‐centre, prospective, observational cohort ‘*Cancer HALLmark Epigenetics aNd Genetics* of BLCA’ (Challenge‐BLCA) with 224 donors enrolled from 5 hospitals across China preserved at a Central China Biobank (Tables S1–S4 and Figure 1).

## 2 MATERIALS AND METHODS

### 2.1 Clinical protocol and ethics statement

This study was conducted in accordance with the measures of China on the administration of clinical research, the Declaration of Helsinki and the Ethic Protocols of Human Genetic Resource Preservation Center of Hubei Province, China (Hubei Biobank). Clinical information and samples (including blood, urine, surgical tissue specimens and primary cancer cells) of the multi‐centre, prospective, observational cohort for BLCA: Cancer HALLmark Epigenetics aNd GEnetics (CHALLENGE) of BLCA (named: Challenge‐BLCA study cohort) was collected from five hospitals in four cities of China: Wuhan, Enshi, Beijing and Panzhihua, treated and preserved by the Hubei Biobank, the official member of the International Society for Biological and Environmental Repositories (https://irlocator.isber.org/details/60), approved by the Ethic Institutional Review Board (approval number: 2015029, 2017038 and 2020102) and China Human Genetic Resources Management Office, Ministry of Science and Technology of China (approval number: 20171793, approved capacity of samples: 2.1 million).

Sample‐size estimation: Genome‐wide DNAm level from MIBC, NMIBC and normal bladder tissue from a previous study^41^ was used to calculate DNAm difference between these tissues. We built artificial ‘urine’ DNA methylome by mixing MIBC/NMIBC into normal tissue data at a 5% percentage. General linear models were built upon these data to classify cancer versus normal or MIBC versus NMIBC. Based on the distribution of these model, we empirically determine the minimal sample size at alpha = .05 and power = .8 to a total of 170, with 120 cancer patients and 50 healthy donors. A drop‐off rate of 25% was included, to make a targeted enrolment of 226.

Written informed consents were obtained from all individuals enrolled in the Challenge‐BLCA study cohort. The samples (*n* = 304) from a total of 224 donors (Table S1) were continually and prospectively collected by the Hubei Biobank as bladder tissue (*n* = 33) from 2018/10/25 to 2020/10/31, urine samples (*n* = 271) from 2018/09/12 to 2020/05/23. Clinical, pathological and follow‐up data records for putative BLCA patients (*n* = 145) and healthy donors (*n* = 79) were collected (Tables S1 and S2), and three pathologists were invited to independently confirm the histology diagnosis for putative BLCA patients. Due to a low clinical application of FISH assay, we collected historical pathology reports of FISH (*n* = 368, from 2015 to 2021) for putative BLCA patients from the participating centres. Routine laboratory tests and pathology assessments were done according to the relevant Chinese clinical guidelines and protocols.

Enrolment, aims and including/excluding criteria for the Challenge‐BLCA study cohort are listed in Tables S1 and S2. The flow chart of the study is depicted in Figure 1.

The training cohort contains 33 BLCA tissues and 12 urines from healthy donors (Table S3). The validation cohort contains 259 urine samples from a total of 210 donors (133 patients and 67 healthy donors, Table S4). Third‐party statistician was invited to blind the samples and perform double‐blinded validation.

### 2.2 Biospecimen collection

RT4, T24 and 5637 cells were kindly provided by Cell Bank, Chinese Academy of Sciences (Shanghai, China) and cultured under identical conditions following standard procedures. Clinical assessment of BLCA was done according to the EAU 2020 Oncology Guidelines (https://uroweb.org/individual‐guidelines/oncology‐guidelines/). Human peripheral blood was collected with BD EDTA tube according to the manufacturer's protocol and stored at 4°C for no longer than 8 h before serum separation. Peripheral blood mononucleus cell (PBMC) was separated with the standard Ficoll protocol. Fresh tumour or normal tissues was collected during surgery and transferred to laboratory in high glucose, 10% FBS supplemented DMEM. Tissue sample was resected in PBS prior to single‐cell dissociation. For methylation sequencing, tissue samples were flash frozen in liquid nitrogen and stored at −80°C. Urine samples were collected in a 50‐ml FALCON tube and being stored at −80°C within 4 h of collection.

### 2.3 RNAi of SOX2

SOX2 siRNAs were purchased from GenePharma (Shanghai, China). The target sequences siRNAs were as in Table S5. Cells were transfected with Lipofectamine 3000 following the manufacturer's instruction (Invitrogen Ltd.).

### 2.4 Cell migration and invasion assay

For migration assay, 1 × 10^5^ 5637 cells or 4 × 10^4^ T24 cells were plated in the upper transwell chamber (Corning Ltd., USA) with 200‐μl serum‐free 1640 medium and 600 μl of 1640 medium containing 10% FBS in the lower chambers to induce cell migration. After incubated for 24 h at 37°C, cells were fixed with 4% paraformaldehyde and stained with .1% crystal violet. The migrated cell numbers were counted using phase contrast microscopy and statistically analysed.

For invasion assay, Matrigel was thawed and liquefied on ice and diluted in cold serum‐free 1640 medium to a final concentration of 200 μg/ml. Next, 100 μl of the diluted Matrigel was carefully added to the transwell insert and solidified in a 37°C incubator for 2 h to form a thin gel layer. Then, 1 × 10^5^ 5637 cells or 4 × 10^4^ T24 cells were plated in the upper transwell chamber (Corning Ltd., USA) with a 200‐μl‐serum‐free 1640 medium and 600 μl of 1640 medium containing 10% FBS in the lower chambers to induce cell migration. After incubated for 24 h at 37°C, cells were fixed with 4% paraformaldehyde and stained with .1% crystal violet. The migrated cell numbers were counted using phase contrast microscopy and statistically analysed.

### 2.5 Western blot

Western blot assay was performed as previously described by Xiong et al. in our group.^49^ The antibodies used in this study were purchased from the following indicated companies: SOX2 (Abcam Ltd., #ab97959), E‐Cadherin (CST Ltd., #3195), *N*‐Cadherin (CST Ltd., #13116), Vimentin (CST Ltd., #5741), GAPDH (Santa Cruz Ltd., #sc‐365062). A detailed list of antibodies could be found in Table S6.

### 2.6 KO of SOX2 and DMR

The small guide RNA (sgRNA) sequences were designed by using the CRISPR Design Tool (http://tools.genomeengineering.org), kindly provided by Feng Zhang lab (Broad Institute of MIT and Harvard, Boston, USA). The target sequences sgRNAs were as in Table S7.

To construct 5637‐Cas9, RT4‐Cas9 and T24‐Cas9 stable cell lines, the Cas9 lentivirus packaging was provided by Ubigene Ltd., Guangzhou, China. The lentivirus particles were used for the infection of target cells with the supplement polybrene. After two rounds of infection, cells were selected with 200‐μg/ml hygromycin.

To establish the cell lines that stably expressed the DMR or SOX2‐KO system, the Cas9 stable cell lines were infected by the DMR or SOX2‐KO sgRNAs lentivirus (Ubigene Ltd.) with the supplement polybrene. After two rounds of infection, cells were selected with 1‐μg/ml puromycin.

### 2.7 Single‐cell ATAC and RNA analysis

We used the integrated scATAC + scRNA object from Xiao et al.^41^ and extracted normal urothelium, luminal‐like, basal‐like and TM4SF1‐positive cancer subpopulation (TPCS) cells from the scATAC object. scATAC peaks were called via MACS2 in ArchR package. *Cis*‐regulatory interaction between chromosomal loci were inferred with ArchR as calculating ‘addCoAccessibility’ (maxDist = 1000 000) and filtered with correlation >.1. Overlapping of scATAC peaks and *cis*‐interacting ‘loops’ to enhancer or DMR are performed with GenomicRanges in R.

### 2.8 CUT&Tag sequencing

RT4, T24, 5637‐Cas9, and 5637‐Cas9‐active‐gDNA cells were counted using trypan blue (Solarbio Ltd., Beijing, China). After quantification, 40 million cells were used for CUT&Tag experiment. CUT&Tag experiments were performed with NovoProtein CUT&Tag 2.0 pAG‐Tn5 kit (NovoProtein Ltd., Cat. #N259) according to the manufacturer's protocol. Antibodies used in this study include anti‐H3K27ac (Abcam Ltd., Cat #ab4729), anti‐FOXA1 (Abcam Ltd., Cat. #ab170933), anti‐CTCF (Abcam Ltd., Cat. #ab188408), anti‐SOX2 (Abcam, Cat. #ab92494), Goat‐anti‐mouse IgG (Sangon Ltd., Cat. #D111024) and Goat‐anti‐rabbit IgG (Sangon Ltd., Cat. #D111018). Each library was sequenced to 2× human genome coverage on NovaSeq sequencer (Illumina Ltd., CA, USA). A detailed list of antibodies could be found in Table S6.

### 2.9 CUT&Tag analysis

Raw paired‐end CUT&Tag sequencing data were mapped to human reference genome GRCh38 using Bowtie2 (‐k 10 –very‐sensitive ‐X 2000) (https://github.com/BenLangmead/bowtie2). All unmapped reads, non‐uniquely mapped reads, reads with low mapping quality (MAPQ) < 20 and PCR duplicates were removed. Enrichment peaks were determined by intersecting peaks found from MACS2 callpeak (‐f BAMPE) (https://github.com/taoliu/MACS) and Genrich (standard parameter) (https://github.com/jsh58/Genrich). Differential peak calling was performed with a general linear model approach for estimating difference.

### 2.10 RNA sequencing

The total RNA of RT4, 5637, 5637‐Cas9, 5637 DMR and SOX2‐KO was extracted using Trizol (Invitrogen Ltd.), and 1000 ng of RNA were taken for ribosomal‐off treatment using the Ribo‐off rRNA Depletion Kit (Human/Mouse/Rat) (Vazyme Ltd., Cat. #N406‐01). RNA library with Ribo‐off was constructed by a KAPA RNA HyperPrep Kit (Roche Ltd., Cat. #KK8544). Each RNA library was sequenced with 150‐bp paired‐end format to 2× human genome coverage on NovaSeq sequencer (Illumina Ltd., CA, USA). All procedures followed the standard manufacturer's protocol.

### 2.11 RNAseq analysis

Raw sequencing data (fastq) were trimmed with fastp (‐w 10) and aligned to GRCh37 reference genome with STAR (–chimSegmentMin 20 –chimScoreMin 5 –quantMode GeneCounts –twopassMode Basic) before re‐alignment with HISAT with the STAR‐output novel splicing sites. Read counts per gene (GRCh37.82 ENSEMBL annotation) were extracted from the HISAT‐output binary alignment format (BAM). Differential expression was performed with DESeq2 with annotation from EnsDb.Hsapiens.v86. Significantly differential expressed genes were defined by padj <.001 and abs(log2FoldChange) >1. Similarities between pairs of samples were computed with Euclidean distance (dist function in R) upon all possible differentially expressed genes padj <.05 and abs(log2FoldChange) >.5.

### 2.12 DNA extraction

For the extraction of gDNA from BLCA tissues and its adjacent tissues, about 25 mg of tissues was taken from each sample and extracted according to the DNeasy Blood & Tissue Kit (QIAGEN Ltd., Cat. #69506). cfDNA in urine was extracted from 20 ml of urine supernatant with Quick‐DNA Urine Kit (Zymo Research Ltd., Cat. #D3061). All procedures followed the standard manufacturer's protocol.

### 2.13 Oncology panel sequencing

DNA was sonicated into ∼250‐bp fragments with Covaris S220. NGS sequencing libraries were built with a single‐stranded DNA ligation protocol. In brief, sonicated DNA was denatured to form a single strand and 3′‐polyA‐tailing was performed with terminal transferase (Enzymatics Ltd., USA, Cat. #P7070). Ligation of a polyT‐extruding adaptor (Sangon Ltd., China) was performed with *Escherichia coli* ligase (Takara Ltd., Japan, Cat. #2161). Linear amplification of the ligated product was performed with adaptor‐specific primer (Sangon Ltd., China) for 12 cycles and the amplified product was annealed and ligated into a 5′‐polyN‐extruding adaptor (Sangon Ltd., China) with T4 ligase (Enzymatics Ltd., USA, Cat. #L6030). The ligated product was then amplified with Illumina‐compatible primers (Sangon Ltd., China) for 10 cycles. The amplified library was captured using a custom‐synthesized oncology panel‐consisting exons, UTR and structural variant breakpoint‐enriched introns of 538 tumour‐related genes, as well as 1076 SNP loci (Euler Technology Ltd., China). Libraries were sequenced to targeting ∼800× on‐target coverage with paired‐end 150‐bp read format on Illumina NovaSeq.

### 2.14 Oncology panel data analysis

Raw sequencing data were mapped using BWA‐MEM to GRCh37 reference genome with default parameters. Germline mutations were called with the Sentieon haplotyper and annotated with VEP (90.1) and SnpSift (4.2). Paired tumour‐normal samples were co‐called and candidate germline variants were filtered with the gnomAD global frequency <.001 and in‐house database frequency <.001 (out of 20 000 patients). Somatic tumour mutations were called with the Sentieon TNscope and Pisces (5.2.9.122), whereas variants called by both algorithms were passed for filtering. Copy number variations were called using a CNVkit with a default parameter. B‐allele frequency (BAF) determination was performed for germline and somatic variants. Tumour genome was segmented using BAF and sequencing depth information. Allelic copy numbers were determined for each somatic variant using a hypergeometrical test. Structural variants were called using Lumpy. Tumour content determined by an in‐house CNV‐based linear regression method was confirmed by haematoxylin and eosin staining. Minimal tumour‐cell‐fraction of 5%/2%, a minimal variant read number of 10 and a minimal read depth of 500 were applied to variants for filtering. Filtered mutations were annotated with vcfanno and filtered with gnomAD global frequency <.001.

### 2.15 DNA methylation data processing

Pre‐processing of genome‐wide bisulfite capture sequencing data is similar as from Xiao et al.^41^ Briefly, raw bisulfite‐converted DNAm sequencing data were processed using fastp and mapped to GRCh37 + decoy reference genome using BWA‐Meth. Mapped data were deduplicated and sorted using Sambamba and Samblaster. CpG‐methylation level was extracted using a Pile‐O‐Meth toolkit. For all libraries, conversion rate was quality controlled by CHH methylation level >99%. Basic statistics of in‐house sequencing library were further quality‐controlled by on‐target rate and on‐target coverage with bedtools, and duplication rate and mapping rate with Sambamba. CpG methylation level (beta: defined as reads of C nucleotide over total read coverage on single C bases on both strands on CpG loci) was measured for each CpG loci across the genome as mentioned earlier using Pile‐O‐Meth. For each locus, beta from sequencing results were summarized in R (3.6.2) using an in‐house script. Differentially methylated loci (DML) were defined as (1) *p* < .01 for *t*‐test between control and case groups (given NMIBC/MIBC or BC/normal urine); (2) beta difference between case and control groups>.1. Initial DMR candidates were made by merging within‐100‐bp‐apart DML. The average beta of each initial DMR was calculated as mean beta of all CpG encompassed in the DMR. This average beta was subjected to *t*‐test and *p* < .01 regions were selected as candidate ‘seed’ DMR. Segments of methylation difference level were computed using a circular binary segmentation approach on beta difference case and control groups with DNAcopy. *k*‐Means clustering was performed using R (3.6.2) on the methylation beta difference on each segment, and clusters of segments fully encompassed candidate ‘seed’ DMR were selected as true DMR candidate. DMR candidates were overlapped with scATAC peaks from luminal, basal, TPCS and UE cells. DMR candidates not overlapping with epithelial cell‐type‐specific scATAC peaks were removed. The leftover candidate DMR set was clustered using *Z*‐normalized beta value and hierarchically clustered, segregated into groups by *k*‐means clustering. Candidate ‘core DMR’ is selected from the candidate DMR set by correlating pathological features to unsupervised hierarchical clustered groups of tumour hyper‐methylated DMR.

### 2.16 Bisulfite‐converted DMR‐specific amplicon sequencing

Tissue genomic DNA (200 ng) or urine total DNA (100 ng) were bisulfite converted using an EZ‐DNAm‐Gold Kit (Zymo Research Ltd., Cat. #D5006) and the DNA is dissolved in NF‐H_2_O. For urine DNA less than 100 ng, λDNA (TaKaRa Ltd., Cat. #3010) was added to a total of 100 ng before bisulfite conversion to reduce DNA damage. After conversion, the DNA is amplified by the multiplex PCR system contained 20‐ng DNA, 2‐μl 10× Buffer II (100‐mM Tris–HCl, pH 8.3, 500‐mM KCl), 1.2‐μl 25‐mM MgCl_2_, .4‐μl 10‐mM dNTPs, 4.6‐μl primer mix, .1‐μl AmpliTaq Gold DNA Polymerase (Thermo Fisher Ltd., Cat. #N8080241), 20‐ng DNA and NF‐H_2_O to a total of 20 μl. Amplification was performed as follows: a denaturation step at 95°C for 10 min, followed by 25 cycles × (95°C for 30 s, 65°C for 30 s, 54°C for 2 min, 65°C for 30 s and 72°C for 30 s), 72°C for 10 min and 4°C for overnight. PCR products were purified with AMPure XP beads. Finally, the PCR products were amplified using adaptor oligos (Sangon Ltd., Shanghai, China) and KAPA HiFi HotStart ReadyMix (Roche Ltd., Cat. #KK2602), resulting in a final amplicon library that was further purified before sequencing by 150‐bp paired‐end format to a target of 6‐M reads on Illumina NovaSeq. A list of primers could be found in Table S8.

### 2.17 Bioinformatic processing of amplicon sequencing data

Raw amplicon sequencing data (fastq) were aligned to GRCh37 + decoy reference genome using Sentieon BWA‐MEM. Sentieon UMI processing pipeline was used to collapse the paired reads as well as UMI groups. After initial alignment, a reference sequence with converted C > T loci denoted was added to the BAM file using PySAM. Reads, with either <99% conversion rate, ≥2 non‐converted C on non‐CpG loci, non‐single‐stranded converted read, both‐strand converted, or MAPQ <30, were removed using PySAM. Haplotype extraction was done with Rsamtools in R. Methylation frequency (mf) of a read is defined as

$$C_{CpG}/N_{CpG}$$

where *C* is the number of C in CpG and *N* is the number of total CpG in the read.

A DNAm haplotype is defined as a read originated from a given amplicon with a specific methylation frequency. To simplify computation, all methylation frequency is rounded to one digit of decimal. For each sample, reads on any given amplicon are aggregated according to the DNAm haplotype. The relative prevalences (‘haplotype frequency’) of the *j*th haplotype on amplicon were defined as

$$p_{j}=\left(N_{haplotype_{j}}/\sum_{i}^{n}N_{haplotype_{i}}\right)$$

where all haplotypes *i*, *j* belong to similar amplicon.

### 2.18 Statistical classification of DMR

For each amplicon‐compassed DMR on each sample, the haplotype entropy index is defined as

$$Ent_{i}=-1\times \sum_{j}^{n}p_{ij}\times \log\left(p_{ij}\right)$$

where $p_{ij}$ denotes the *j*th haplotype of the *i*th amplicon.

Student's *t*‐test was used to measure any difference between entropy indexes of two groups of samples: MIBC and NMIBC tissues. *p*‐Values were adjusted by false discovery rate (FDR) method. DMR with adjusted *p* ≥ .05 was defined as T1DMR, and DMR with adjusted *p* < .05 was defined as T2DMR.

### 2.19 Association of methylation haplotypes to traits

Student's *t*‐test was used to measure any difference between haplotype frequencies from two given sets of samples: HG/MIBC (100% HG) cancer versus LG/NMIBC (mostly LG) cancer tissue, HG cancer tissue versus normal urine, LG cancer tissue versus normal urine. *p*‐Values were adjusted by an FDR method. Haplotypes with adjusted *p* < = .001 and at least a haplotype frequency of 10% in the higher group were considered significantly different between the two groups. The haplotypes were grouped into three different classes: high in HG cancer (relative to urine and LG) (*HG_high*), high in LG cancer (relative to urine and HG) (*LG_high*) and high in urine (relative to MIBC and NMIBC) (*Urine_high*). Mean haplotype frequency of all haplotypes from the same class was computed for each sample. From the results, we consider that more reliable biological classification of BLCA might rely on the WHO grade as well as clinical stage instead of the highly random muscle invasion manifested on pathological slides.

### 2.20 Tissue‐of‐origin decomposition

Mean haplotype frequency of each haplotype was computed for three sets of samples: HG cancer tissues, LG cancer tissues and normal urine. The nnls function from R package lsei was used to compute a non‐negative least‐squared decomposition of the sample into a mixture of previously mentioned three different tissue‐of‐origin components: *LG_similarity, HG_similarity and Urine_similarity*.

### 2.21 LoD of amplicon sequencing

Standard samples were prepared by mixing a defined portion of cancer cell line or PBMC genomic DNA into urine cfDNA. Different mix‐in gDNAs (RT4, T24, 5637 and PBMC) were mixed with cfDNA from normal urines from individual donors of the same sex, respectively. At least two different samples of normal urine cfDNA were used for a given mix‐in gDNA. Serial dilution was performed to make 100%, 20%, 10%, 5%, 2.5%, 1%, .5% and .1% concentrations of mix‐in gDNA in urine cfDNA. The standard curve samples were then subjected to bisulfite conversion, multiplex amplification and sequencing according to the respective protocol. Haplotype frequencies were extracted from the processed sequencing result. Prediction was done with the urine cancer score (UCAS) model. Limits‐of‐detection were drawn to 2×, the point which the next lower dilution is statistically indifferent to the current dilution by Student's *t*‐test.

### 2.22 Classifier training

Two different general linear models were trained to classify amplicon sequencing data. The UCAS model was trained to distinguish cancer tissue from normal urine, using a Gaussian family distribution and a link function of the following:

$$\begin{array}{ccc} & & Predictor∼Urine\_high+HG\_high+LG\_high\\ & & \;+\;Urine\_similarity \end{array}$$

where Urine_high, HG_high and LG_high are mean frequency of denoted classes of haplotypes as defined by *t*‐test, and Urine_similarity is the non‐negative least squared approximation of ‘urine‐like’ mixture component in sample. Predictor==1 when the sample is a cancer tissue or ‘==0’ otherwise.

The BLCAS model was trained to distinguish HG cancer tissue from LG cancer tissue, using a Gaussian family distribution and a link function of the following:

Predictor∼HG_high+LG_high

where HG_high and LG_high are mean frequency of denoted class of haplotypes as defined by *t*‐test. Predictor==1 when the sample is an HG cancer tissue or ‘==0’ otherwise.

Training was done with 32 urothelial cancer tissue samples (excluding the neuroendocrine cancer sample) and 12 normal urine samples, from a total of 14 MIBC, 9 NMIBC and 12 healthy donors. With the training group, cut‐off of UCAS was set to .42 and BLCAS was set to .52.

### 2.23 Clinical trait association and statistical analysis

Clinical trait was compared to the UCAS and BLCAS results in a double‐blinded fashion. Progression‐free‐survival was defined as the time between first operation and subsequent re‐surgery or clinical assessment and right‐censored. Survival analyses were done with R package survival. Hazard ratio and statistical significance tests were done with Cox model (R function *coxph*) and statistical comparison was done by log‐rank test. Survival plots were done with R package survplot. ROC, AUC and best performance sensitivity/specificity were calculated with R package pROC. For table analysis, the Fisher test was used. For comparing prediction precision, *F1.test* with R package MLmetrics was used where applicable. Unsupervised hierarchical clustering was done by R function *hclust* with a Ward method and classification according to hierarchical clustering result was done with *cutree*.

### 2.24 Software

A list of software used in the study could be found in Table S9.

## 3 RESULTS

### 3.1 The challenge‐BLCA cohort

We screened 879 patients suspected of having BLCA and planned to undergo surgery, and 100 healthy donors from 5 hospitals across China, to prospectively recruit 145 patients and 84 healthy donors into the Challenge‐BLCA cohort (Figure 1). The primary aim of this study is (a) to classify and validate the biological nature of BLCA‐associated DNAm regions; (b) to determine the utility of DNAm on tumour‐specific DMR as biomarkers for non‐invasive detection and surveillance of BLCA. Specifically, we sought to determine whether such an approach results in precision biomarkers which facilitate (1) the detection of cancer in pre‐surgery urine from BLCA‐history naïve patients; (2) the prediction of the presence of HG cancer from pre‐surgery urine; (3) the prediction of tumour pathology from pre‐surgery urine; (4) the prediction of progression‐free survival and metastasis from pre‐surgery urine; (5) the prediction of the presence of residual cancer from paired pre‐ and post‐surgery urine; and (6) the prediction of recurrence or residual disease with post‐surgery urine.

The training cohort (tumour samples from 23 patients and urine from 12 healthy donors) was applied to develop the BLCA‐specific DNAm statistical model. In contrast, the validation cohort (259 urine samples from 135 patients and 67 healthy donors) was used to validate the non‐invasive detection assay.

The 23 BLCA patients in training cohort were with a skewed sex ratio (male:female = 20/3, 6.67:1) and comprised all possible clinical grades (Ta: 7, 30%; T1: 2, 9%; T2: 6, 26%; T3: 5, 22% and T4: 4, 13%) as well as pathological grading (low: 6, 26%; mixed: 1, 4% and high: 16, 70%). The 133 patients in the validation cohort also had a skewed sex ratio (male:female = 110/23, 4.78:1). They were diagnosed either by post‐enrolment surgery (BLCA‐naïve: 85, 63.9%) or pre‐enrolment surgery (with a BLCA history: 48, 36.1%). These patients were with typical BLCA (109, 82%), atypical BLCA (10, 7.5%), BLCA with multiple synchronous cancer (5, 4%) and LG PUNLMP (5, 4%) or inflammation (4, 3%). After enrolment, pathology after surgery resection determined T stage in 86 (64.6%) of the patients, with 27 (20.3%) Ta, 30 (22.6%) T1, 17 (12.8%) T2, 5 (3.8%) T3 and 7 (5.3%) T4 tumours. We conclude that the study cohort was well balanced and representative of clinically observed BLCA cases.

### 3.2 Statistical classification of BLCA‐associated methylated regions

**FIGURE 2:**
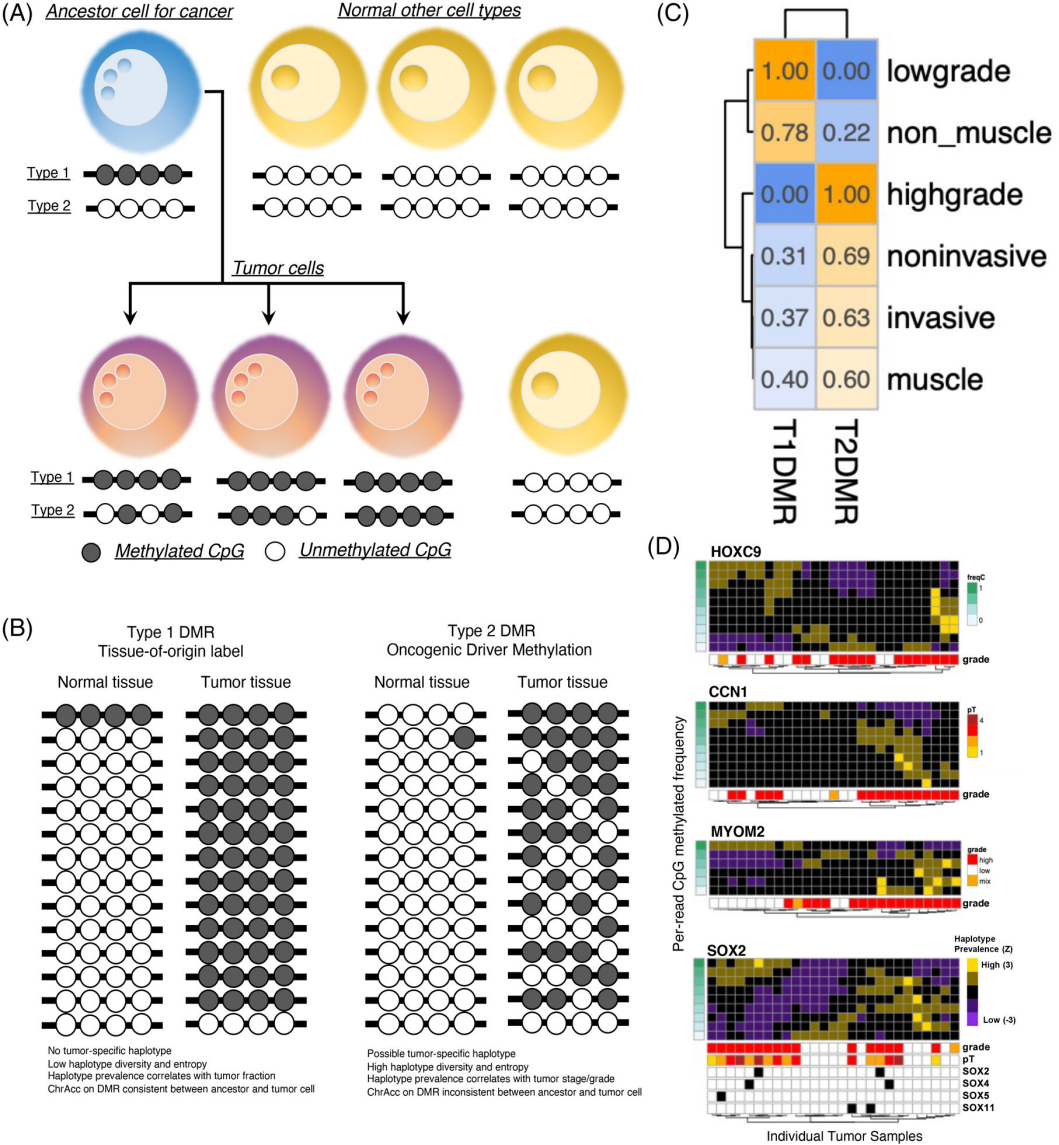
Classification of bladder cancer (BLCA) differentially methylated region (DMR). (A) Cancer‐associated DMR could result from four different scenarios: (1) inherited cell‐type‐specific DNA methylation (DNAm) signature from the clonal ancestor of cancer, which is a normal cell (Type I, T1DMR); (2) de novo DNAm shift during oncogenesis (Type II, T2DMR); (3) inherited cell‐type‐specific DNAm signature from a non‐cancer cell which is absent in normal tissue (Type III, T3DMR), such as immune cells; and (4) de novo DNAm shift accompanied oncogenesis in a non‐cancer cell type (Type IV, T4DMR), such as reprogrammed fibroblast. Considering only the cancer‐cell‐derived DNAm, two types of cancer‐cell‐associated DMR might present: the T1DMR, which is present in the ancestral cell of cancer, and T2DMR, which underwent tumour‐specific DNAm change. (B) T1DMR does not contain tumour‐specific haplotype (TSH) and shows low haplotype diversity. Chromatin accessibility on T1DMR does not change between ancestral cells and cancer. But T1TSH prevalence is highly correlated with tumour fraction in tissues. In contrast, T2DMR exhibits that high haplotype diversity has TSH and shows chromatin accessibility change. Furthermore, T2TSH prevalence is linked to tumour grade or clinical stage. (C) Association of haplotype from T1DMR/T2DMR with pathological traits. (D) Examples of four T2DMR that display oncogenesis‐associated de novo methylation/demethylation, as revealed by gradually changing TSH prevalence in tumour samples.

We term the mean cytosine methylation status on multiple CpG islands in a single‐DNA read as a *methylation haplotype* (haplotype in short). Theoretically, haplotype in T1DMR is unchanged during oncogenesis, whereas haplotypes in T2DMR occurred de novo in cancer cells and might be associated with tumour grades or stages (Figure 2A). DNAm on T1DMR loci does not hold oncogenic capability but is passively amplified during tumourigenesis. In contrast, DNAm on T2DMR is associated with or directly driving, malignancy transformation. We term T1DMR as ‘passenger’ and T2DMR as ‘driver’ loci.

We reasoned that the classification of T1DMR and T2DMR could be performed with haplotype diversity (entropy), prevalence, as well as the existence of tumour‐specific haplotype (Figure 2B). In theory, haplotype diversity from T1DMR should be significantly lower compared to T2DMR, and tumour‐specific haplotype prevalence in T1DMR should be higher compared to T2DMR. Furthermore, tumour‐associated haplotypes from T1DMR should be present in normal tissues, albeit at a lower level, whereas those from T2DMR should be near absent.

By associating pathology‐deduced tumour fraction to differential methylation levels, we located 17 BLCA‐associated DMR from our previous study^41^ as potential T1DMR or T2DMR. To saturate sample the haplotypes in BLCA tissues, a multiplex PCR amplicon assay designed to target these 17 DMR was applied to 33 BLCA tumour tissue samples from the 23 donors of the training cohort. Haplotype prevalence and entropy (Figure S1) on each DMR clearly classified T1DMR from T2DMR. The entropy (‘diversity’) of haplotypes on T1DMR does not differ between LG and HG tumours (Figure S1). However, the type of haplotypes on T1DMR drastically differs between LG and HG tumours. For example, OTULINL T1DMR is hypomethylated (>80% haplotype with 0% methylation) in NMIBC but hyper‐methylated (>50% haplotype with 100% methylation) in MIBC (Figure S1A). LG tumour and non‐muscle‐invasiveness are only associated with haplotypes in T1DMR, suggesting that LG tumour results from clonal expansion of a cell type remained in a well‐differentiated status. Haplotypes in T2DMR are associated with HG tumour as well as invasiveness (Figure 2C). Unsupervised hierarchical clustering of haplotype prevalence from each sample (‘trees’ in Figure 2D) revealed that HG tumours cluster together according to the prevalence of haplotypes on T2DMR. In these regions, although LG tumours typically have a defined set of haplotypes, the diversity of haplotypes in HG tumours is significantly higher. Furthermore, gradual methylation or demethylation was found in HG tumour samples (Figure 2D), indicating more prevalent epigenetic reprogramming in HG tumour compared to LG. These T2DMR controls genes known to be implicated in oncogenesis such as HOXC9,^50^ CCN1,^51^ SOX2 or reported being differentially expressed in epithelial cancer such as MYOM2.^52^ Similar to our previous report,^41^ we observed that haplotypes from T1DMR differ between LG and HG tumours, suggesting that they are of different cellular origin.

### 3.3 Functional validation of SOX2‐associated driver T2DMR

To validate that epigenetic reprogramming on ‘driver’ T2DMR is associated with oncogenesis, we investigated a set of T2DMR adjacent to a well‐known oncogene SOX2,^53^ which shows gradual de novo DNAm in HG but not LG cancer (Figure 2D). Our previous works with single‐cell RNA and ATAC sequencing have demonstrated that NMIBC and MIBC evolve from different cell‐of‐origin. Although NMIBC (luminal‐like BLCA) originates from the intermediate cells of the urothelium, MIBC (basal‐like BLCA) originates from the basal cells of urothelium.^41^ Particularly, a TPCS arises in MIBC as a potential cancer stem cell. We leveraged the same single‐cell ATAC dataset to extract the regulatory elements and their chromatin accessibility around SOX2 loci. Furthermore, we applied the CUT&Tag assay on BLCA cell lines T24 (which is derived from an MIBC donor and demonstrates typical basal‐like HG tumour characteristics), 5637 (which is derived from an MIBC donor and shows transcription similarity to TPCS) and RT4 (which is derived from an NMIBC tumour, and representative to an LG tumour) to investigate transcription factor and chromatin regulator binding on this region. scATAC and CUT&Tag experiments revealed two distal regulatory regions (L1 and L4) specifically bound by FOXA1 and opened in the basal cancer cell types (basal and TPCS) (Figure S2A). Correlation between single‐cell chromatin accessibility suggests that L1 and L4 enhancers interact with the SOX2 transcription start site (TSS) (Figure S2A). The chromatin *cis*‐regulatory interaction is supported by basal‐specific CTCF binding on L1 and luminal‐specific CTCF binding on L4 (Figure S2B). FOXA1 binding on L1/L4 in basal cancer cells is associated with H3K27ac disposal (Figure S2A). Hence, basal‐specific FOXA1 binding removed a non‐enhancer *cis*‐interaction between SOX2 and L4, while activating the L1 enhancer and facilitating L1‐SOX2 interaction.

SOX2 T2DMR interacts with L1 enhancer as inferred by scATAC correlation (Figure S2A). We thus adopted CRISPR technology to remove the T2DMR region and investigate their potential regulatory effects on SOX2 expression. Cas9‐mediated removal of T2DMR in TPCS results in a loss of CTCF binding on L1. Additional loss of CTCF binding adjacent to SOX2 (L2/L3) and reversal of CTCF binding on L4 is detected in TPCS with DMR2 removal (Figure S2B), suggesting that SOX2 T2DMR interaction with L1/L4 is essential for the regulatory activation of SOX2. Consequently, the loss of interaction between the SOX2 locus and the L1 enhancer by SOX2 T2DMR removal reduced SOX2 RNA expression (Figure S2C) and subsequently reduced SOX2 binding on its cognate TFBS (Figure S2D). Though SOX2 expression and binding reduction are only partial in SOX2 T2DMR KO, it is sufficient to cause a global transcription switch to phenocopy SOX2 KO (Figures S2E and S3). As knock‐down of SOX2 results in reduced migration and invasion capabilities in the TPCS cell line (Figure S4), these results indicate that SOX2‐associated T2DMRs are essential regulatory elements for SOX2 in basal BLCA and suggest that differential methylation on T2DMR drives oncogenic transformation.

### 3.4 DNA methylation on T1DMR/T2DMR enables accurate classification of BLCA

**FIGURE 3:**
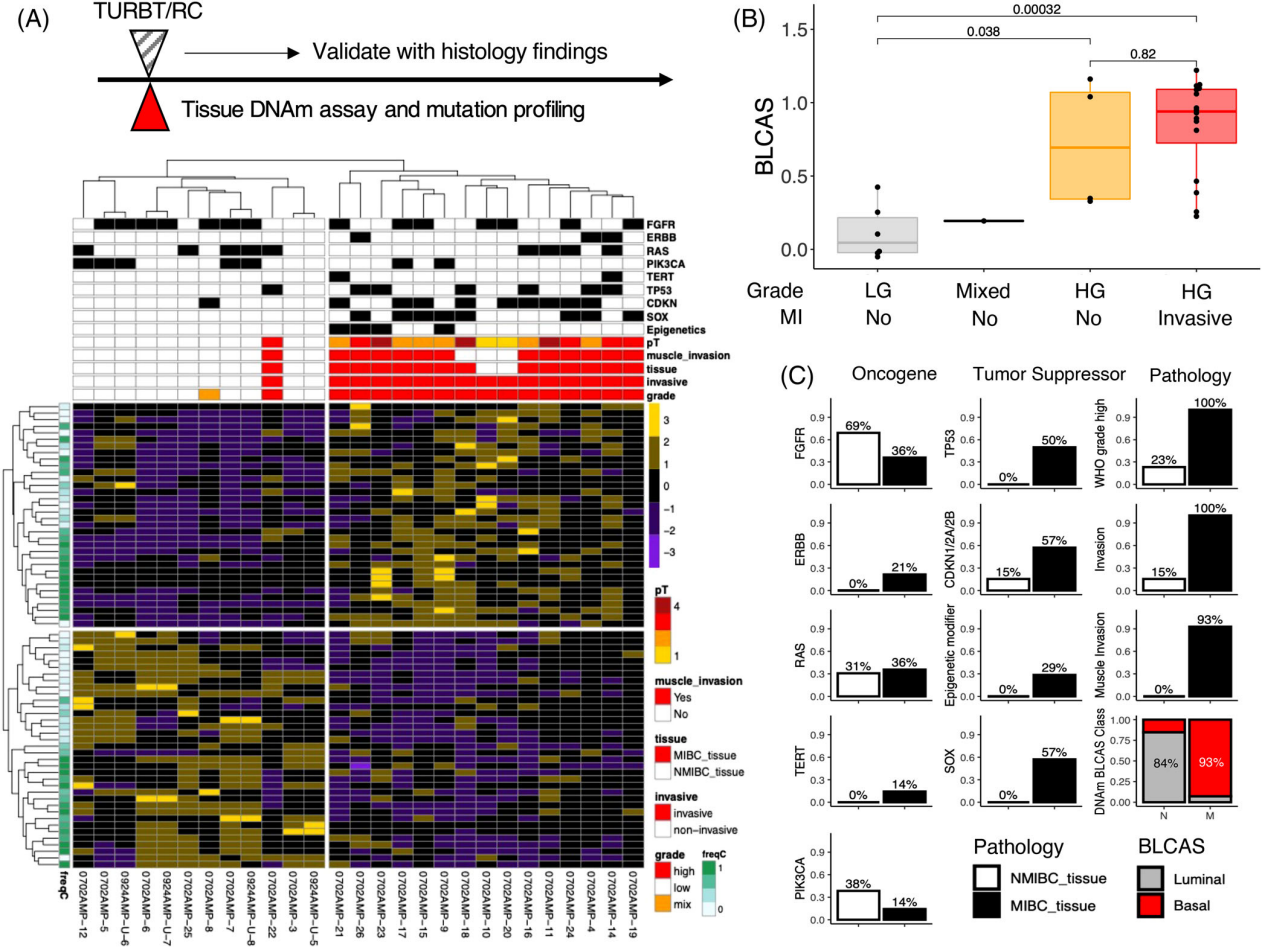
Selective driver and tissue‐of‐origin DNA methylation signature outperform tumour genomic mutation in classifying bladder cancer (BLCA) tumour tissues. (A) DNA methylation and mutation profiling on resected non‐muscle‐invasive bladder cancer (NMIBC) or muscle‐invasive bladder cancer (MIBC) tumour tissues showing haplotype (rows) prevalence in pathologically defined tumour samples (columns). Haplotype prevalence is *Z*‐scaled. Pathological classifications (grade, T‐stage, invasiveness [invasive] and muscle‐invasion) and DNA mutations of known BLCA‐associated oncogenes and tumour suppressors are revealed in the heat map. DNA methylation haplotype prevalence strongly correlates with pathological grade and invasiveness in tumour tissues. (B) DNA methylation‐based BLCAS classifier score predicts tumour grade in MIBC and NMIBC tissues. (C) The presence of mutations or pathology features, or DNA methylation class (luminal or basal, defined by haplotype prevalence), in NMIBC and MIBC.

We tested whether BLCA can be accurately classified with DNAm on the selected driver and passenger T1DMR/T2DMR. MIBC and NMIBC tissues were sequenced using the EUCAS assay, and DNAm haplotypes were extracted for T1DMR/T2DMR. The prevalence of every haplotype was plotted as a heat map by samples. The haplotypes and samples were subjected to unsupervised hierarchical clustering. Haplotype prevalence from these DMRs clearly segregates BLCA into two groups: a luminal‐like group which is associated with LG, non‐invasive phenotype, Ta stage; and a basal‐like group which is associated with HG, invasiveness and higher clinical stage (Figure 3A). The HG group tumours were associated with tumour suppressor mutation (TP53, CDKN and SOX genes), whereas oncogenic mutations were found in both LG and HG tumours (Figure 3A). Based on the tissue classification result, a general linear model was constructed with haplotype prevalence to classify MIBC tissues from NMIBC tissues (Section 2). Numerical results predicted by the model (*BLCAS: basal/luminal cancer score*) clearly stratified MIBC and NMIBC tissues (Figure 3B). BLCAS is strongly associated with WHO grade (*p* = .038 [LG vs. HG MI^−^] or .00032 [LG vs. HG MI^+^], Wilcox test, Figure 3B) but not muscle invasion (*p* = .82 between HG MI^+/−^), showing that malignant transformation predetermined pathological trait before its development. Although mutations on oncogenes or tumour suppressors are associated with pathological grading and clinical features (Figure 3C), their performance is not as good as DNAm‐based classification (Figure S5). Hence, DNAm on the selected DMR accurately reflects the underlying pathological and clinical features of cancer biology.

### 3.5 Non‐invasive detection of BLCA from pre‐surgery urine

BLCA cells release their DNA content into urine at their death. By successfully identifying the driver and passenger DMR containing tumour‐derived DNAm haplotypes associated with BLCA biology, we tested whether the cancer‐associated methylation signature could be identified in urine. In a model experiment, we artificially mixed the genomic DNA from a cancer cell or PBMC (to mimic haematuria) into cfDNA from the urine of healthy donors. DNAm haplotypes in these mixture samples were inferred by sequencing with the same multiplex PCR NGS assay. A general linear model classifying tumour tissues against normal urine was constructed with the methylation haplotype prevalence from sequencing results (Section 2) to predict a score for tumour (*UCAS*). The assay can distinguish samples with 0% and .025% mixed‐in tumour cells, which means its limit of detection is of <.5% (2 × .025%) for the artificial mixture samples (Figure S6). These results indicate that cancer methylation signals could be identified in urine.

**FIGURE 4:**
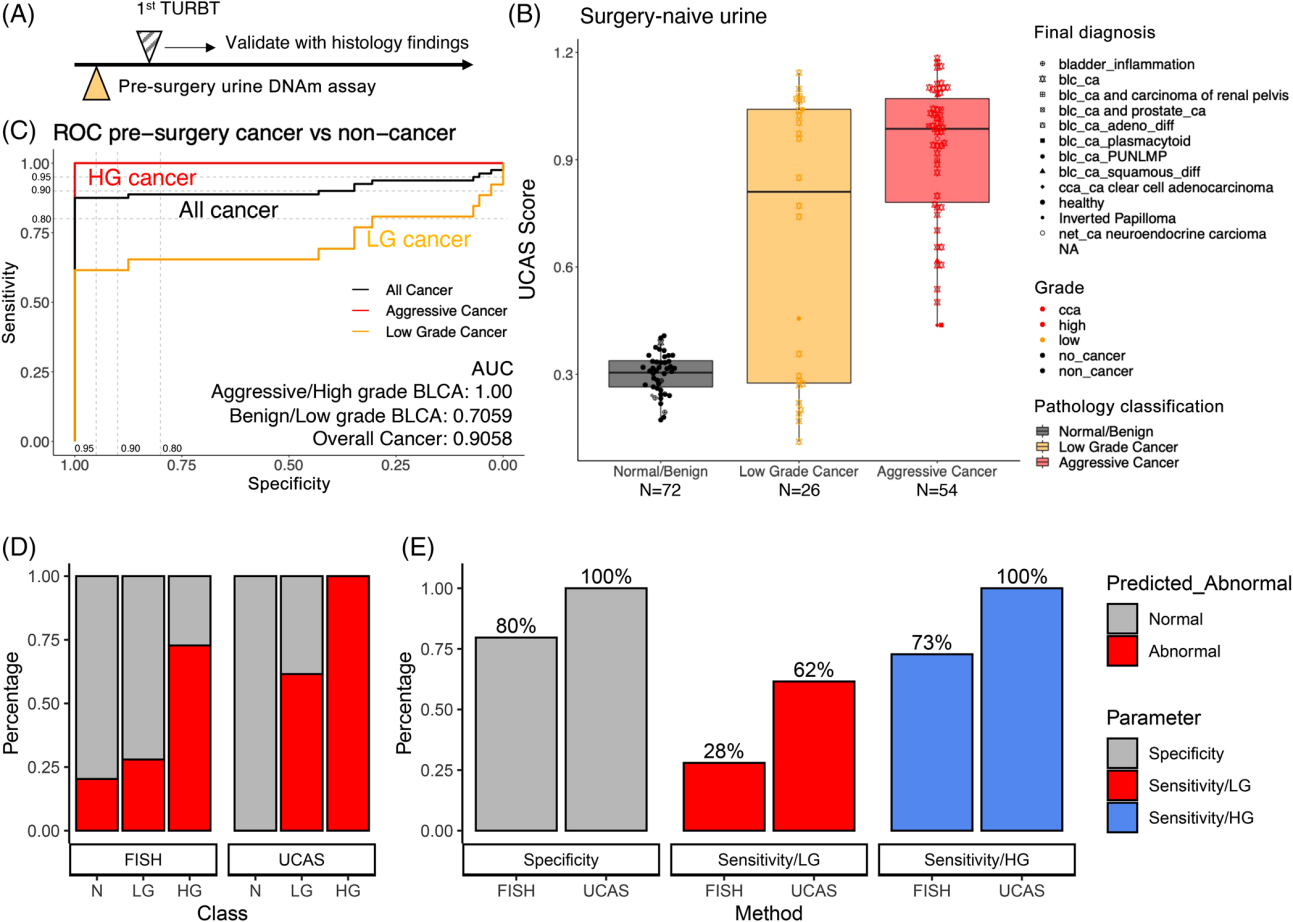
Urine DNA methylation signal non‐invasively detects bladder cancer (BLCA). (A) Experiment design: DNA methylation assays were performed on pre‐surgery urine and validated with resected pathology classification. (B) Cancer‐specific methylation score (cancer methylation score) of individual samples. Samples are grouped/coloured by their class (normal, LG and HG). The individual pathological type of each sample is denoted as the shape of a dot. (C) Receiver‐operating curve and area‐under curve for DNA methylation signature to classify high‐grade (HG), low‐grade (LG) or all cancer samples from benign bladder disease and normal donors. (D and E) Sensitivity and specificity of urine sedimentary cell FISH assay (FISH) or DNA methylation assay (urine cancer score [UCAS]) for LG and HG cancer.

We validated the urine DNAm sequencing assay using the urine samples from the Challenge‐BLCA cohort. In 85 pre‐surgery urine from donors without previous BLCA history and 67 urines from healthy donors (Figure 4A), negative UCAS results were obtained from 100% (72/72) of the urines from healthy donors (67) or donors with benign bladder disease (5). In contrast, positive UCAS results were obtained from 65% of Ta cancer (15/23), 94% of T1 (30/32) and 100% of T2^+^ cancer (25/25) (Figure 4B and Table S10). ROC analysis demonstrated that the AUC for HG BLCA is 1.0, with specificity and sensitivity reaching 100%. The overall detection rate in all the tested samples was 87.5% at 100% specificity, with all undetected tumours as LG (Figure 4C). Compared to historical urine sedimentary cell FISH assay results from the same hospitals, UCAS demonstrated significantly superior sensitivity and specificity for BLCA. For non‐cancerous urine samples, specificity of UCAS is 100% (72/72), whereas specificity for FISH is 80% (47/59) (HR = Inf [3.97, Inf], *p* = 3.529e − 05, Fisher's exact test). For LG and HG tumours, UCAS demonstrated higher sensitivity compared to FISH (LG: 62%, 16/26 vs. 28%, 33/118, HR = 4.08[1.56, 11.17], *p* = .002349, Fisher's exact test: HG: 100%, 54/54 vs. 73%, 139/191, HR = Inf [5.02, Inf], *p* = 6.849e − 07, Fisher's exact test) (Figure 4D,E). The false‐negative urines from LG tumours demonstrated less cancer‐specific T2DMR signal, suggesting that these LG tumours are benign neoplasm resulting from clonal expansions of a minimally transformed cell type (Figure S7). Together, these results suggest that UCAS outperforms currently applied clinical assays to detect BLCA non‐invasively.

In 75 urines from putative BLCA donors who received surgical resection after urine collection and were diagnosed as either HG tumour or tumour‐free, UCAS score was positive for 100% (60/60) of the donors whose first resection was positive for HG tumour. In the remaining 15 urines from patients whose first *trans*‐urethral resection of bladder tumour (TURBT) pathology reports were negative for tumour, a positive UCAS signal was found in 0% (4/4) of donors whose second TURBT pathology remained cancer‐free, and 45% (5/11) of donors whose second TURBT pathology was tumour‐positive, suggesting a positive correlation between tumour load and urine DNAm signal (Figure S8).

### 3.6 Stratification of BLCA patients according to pre‐surgery urine DNAm signature

The BLCAS model was validated by comparing pre‐surgery urine and pathology results from resected tissues from 67 donors with BLCA. Association of BLCAS to resected tumour pathological features suggested that BLCAS is strongly associated with WHO grade and invasiveness but only mildly associated with muscle invasion, with the optimal performance in predicting HG tumour (AUC: .826, Figure S9).

**FIGURE 5:**
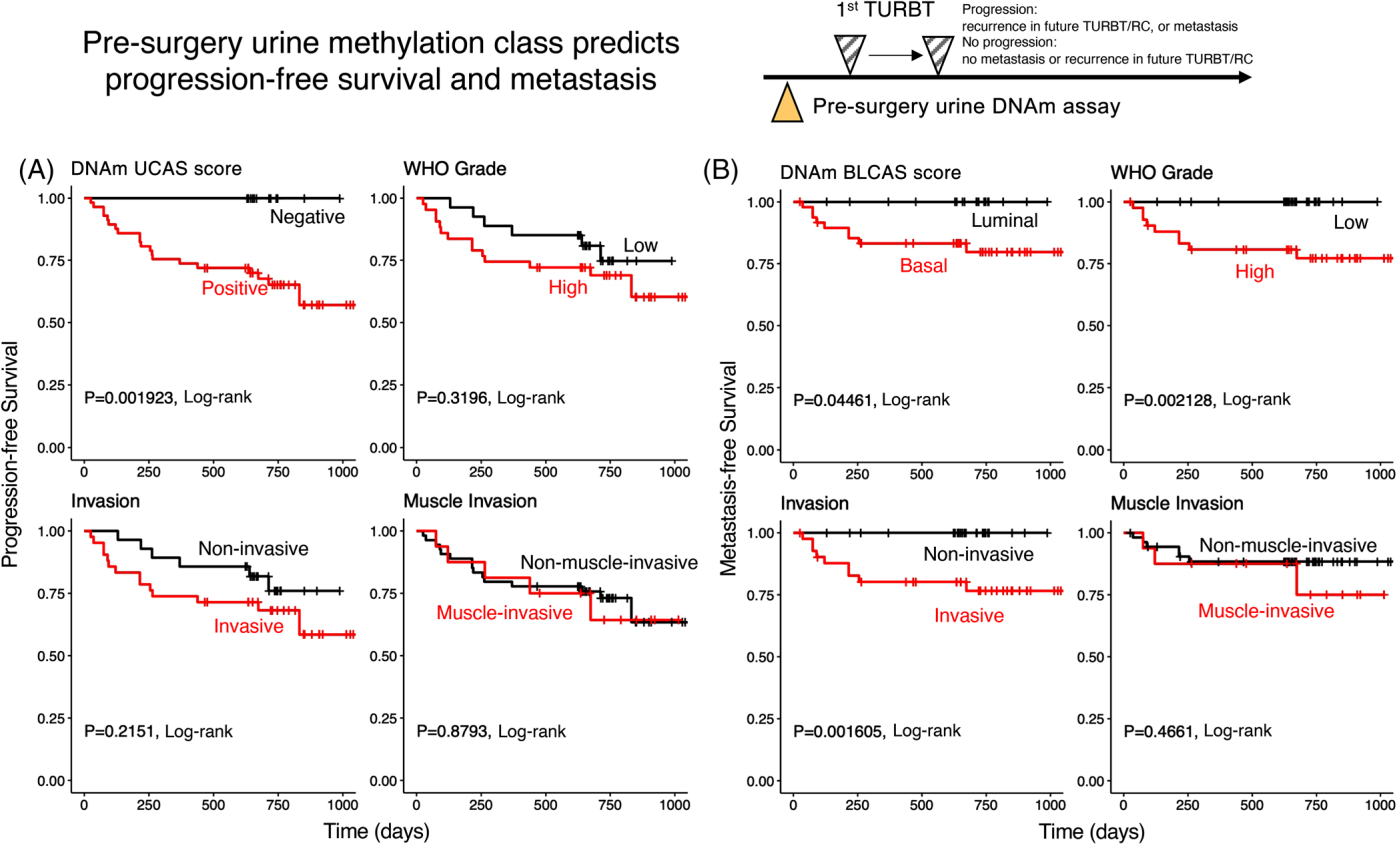
Pre‐surgery urine differentially methylated region (DMR) DNA methylation signal non‐invasively classifies bladder cancer (BLCA) and predicts progression‐free survival. Experiment design: pre‐surgery urine DNA methylation signal, or pathological features of resected samples on the first *trans*‐urethral resection of bladder tumour (TURBT) sample, was used to predict progression‐free survival (A) or metastasis‐free survival (B). Only DNA methylation scores negativeness, but none of the pathological features were significantly associated with a progression‐free survival benefit. DNA methylation, WHO grade and tumour invasiveness, but not muscle invasion, are associated with metastasis‐free survival. In other words, pre‐surgery, BLCA DNA methylation signature negative urine is from benign, slow‐growing cancer, which is very unlikely to develop disease recurrence, and pre‐surgery BLCA DNA methylation basal class are associated with tumours with metastasis potential.

Follow‐up clinical assessments on 70 BLCA donors across 3 years were compared to pre‐surgery urine UCAS and BLCAS scores and pathological tumour reports from the first resection. Progression of disease was defined as tumour recurrence in a second surgery or by clinical assessment. UCAS stratified patients into ‘negative’ or ‘positive’ groups or by BLCAS into ‘basal’ and ‘luminal’ groups. In concordance with the association of UCAS negativity to tumour‐free or well‐differentiated LG tumour state, 100% (13/13) of UCAS negative patients are recurrence‐free, whereas 37% (21/57) patients with positive urine DNAm signal demonstrated disease progression (Figure 5A and Table S11), including patients whose initial TURBT pathology was tumour free. However, 7 months later, TURBT pathology demonstrated LG tumour. Hence, pre‐surgery UCAS accurately stratifies patients into a low‐risk group who were tumour free in a subsequent TURBT surgery (*p* = .001923, log‐rank) and a high‐risk group with an estimated 1‐ and 2.5‐year recurrence rate of 24.6% (95% CI: 12.5%–34.9%) and 43% (95% CI: 25.3%–56.5%) (Figure 5A and Table S11). This stands in stark contrast with all pathological traits tested, including WHO grade (*p* = .3196), tumour invasiveness (*p* = .2151), or muscle‐invasion (*p* = .8793), which failed to predict recurrence‐free survival (Figure 5A and Table S11). Moreover, lymph node or distal metastasis was only found in patients with HG tumour (*p* = .002128), invasive pathology (*p* = .001605) and ‘basal’ class by BLCAS (*p* = .04461). Muscle invasion in pathological assessment failed to distinguish metastasis (*p* = .4661, Figure 5B and Table S11). These results show that urine DNAm signal accurately predicts disease‐free survival to avoid unnecessary additional surgery and stratifies patients of higher risk for metastasis.

### 3.7 Post‐surgery urine DNAm signature detects residual disease and predicts disease progression

**FIGURE 6:**
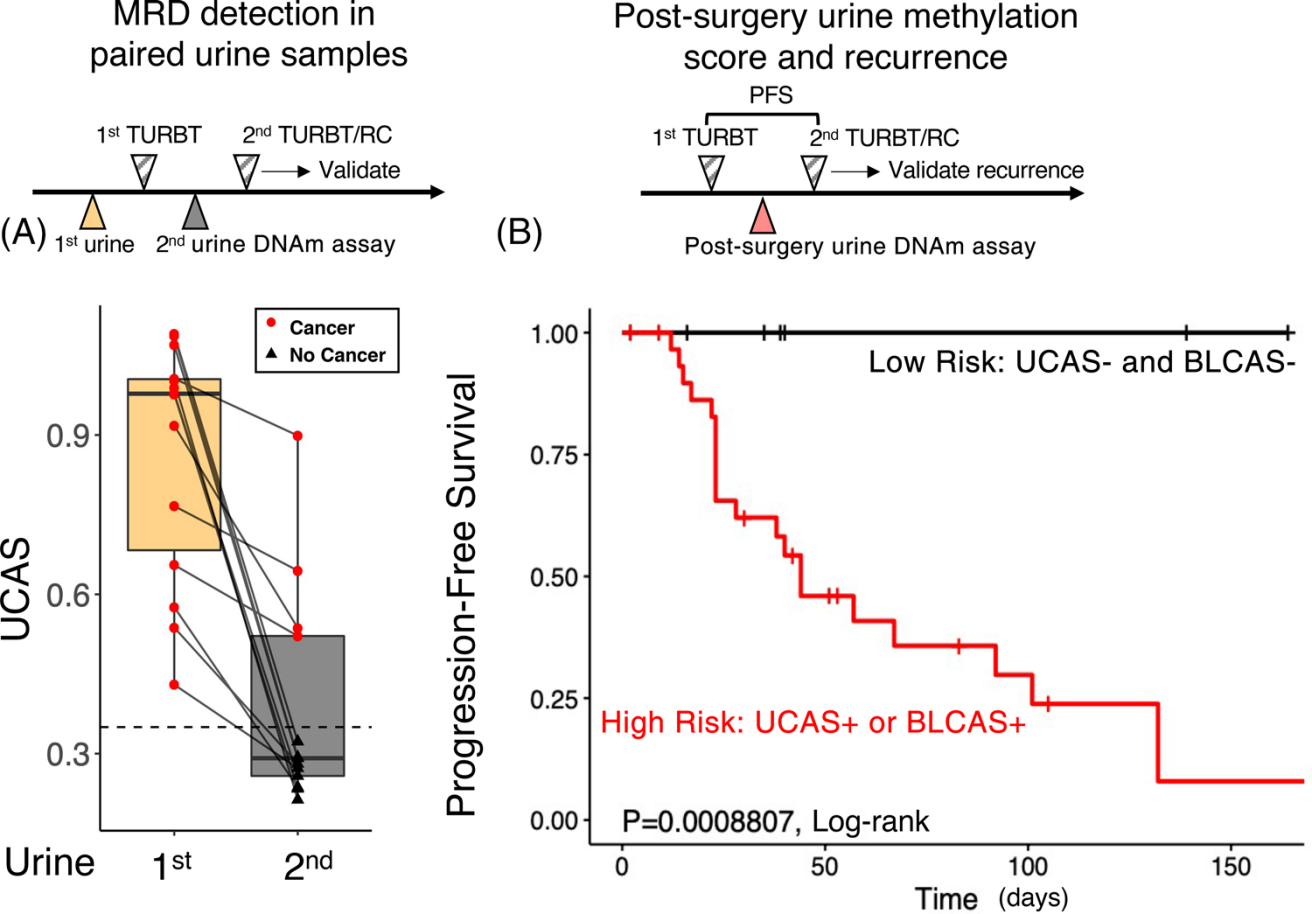
Post‐surgery urine DNA differentially methylated region (DMR) methylation signal detects minimal residual disease and predicts recurrence. (A) Paired pre‐first *trans*‐urethral resection of bladder tumour (TURBT) urine and pre‐second surgery urine are compared for the same individual. Although the pre‐first TURBT urine is 100% positive (13/13) in these patients, second TURBT/radical cystectomy (RC) only finds residual tumour in 100% of individuals (4/4) who show positive cancer methylation score. (B) Post‐first TURBT urine DNA cancer methylation signature (high‐grade or residual disease: high risk; low‐grade and no‐residual‐disease: low risk) stratifies patients into risk groups that are strongly associated with recurrence. A 100% (30/30) of low‐risk patients do not show recurrence within 180 days of the first TURBT, whereas >90% (30/31) of high‐risk patients show recurrence within 180 days.

To determine whether urine DNAm signal could predict residual disease, we first analysed paired pre‐ and post‐first‐surgery urine from 13 donors who received two consecutive surgeries. A 100% (13/13) of first (pre‐first‐surgery) urine was positive for UCAS, whereas a positive second (post‐first‐surgery) UCAS correctly predicts 100% (4/4) of patients who depicted a residual tumour in a subsequent surgery, and 100% (9/9) of patients who were tumour‐free in subsequent surgery were UCAS negative in the post‐surgery urine (Figure 6).

In 61 post‐first‐surgery urines from donors who received two consecutive surgeries within 180 days, we define a high‐risk group with positive UCAS or basal‐like BLCAS and a low‐risk group with luminal‐like BLCAS as well as negative UCAS. Overall, 100% (30/30) of low‐risk group patients are progression‐free within this period, whereas 71% (22/31) of high‐risk group patients depicted residual disease in a second surgery, with an expected disease‐free survival of 44 days (95% CI: 30.4%–69.3%). Hence, the post‐surgery urine DNAm defined high‐risk group shows a significantly higher risk of disease progression (*p* = .0008807, log‐rank). Metastasis is only found in the high‐risk group (6.4%, 2/31) though statistically insignificant due to a low number of cases. These results display that post‐surgery urine methylation signal sensitively detects residual disease and stratifies patients into risk groups with direct implications for clinical decisions.

## 4 DISCUSSION

Our assay demonstrated specificity and sensitivity, reaching 100% for HG BLCA, largely outperforming FISH, urine cytology and the currently available urinary‐based biomarkers.^4^, ^54^ For LG BLCA, our results indicate non‐inferior performance to UtMeMA and superior performance to all the other methods.^40^ Our assay detected all recurrent LG tumours, and all LG tumours with a negative UCAS signal in urine belong to the benign neoplasm group with a favourable clinical outcome. By combining UCAS and BLCAS classifiers, our assay first indicated that urine DNAm could be used to classify patients before surgery. Compared to histology or FISH, the classification of BLCA by pre‐surgery urine DNAm more accurately identifies patients at a high risk of disease progression with negative pathology findings and patients with no risk of recurrence or metastasis. One of the reasons for this might be a full representation of intratumoural heterogeneity in urine DNA compared to limited sampling by pathology.^55^, ^56^ Furthermore, the recurrence of BLCA could occur for transformed LG tumours as well as HG basal tumours. Although histological assessment could distinguish a tumour's grade and invasiveness, it cannot tell the difference between the transformation status of LG tumours, which could only be assessed through molecular profiling. Combining UCAS and BLCAS scores in post‐surgery urine predicts recurrence within 180 days with excellent sensitivity (100%), assisting in stratifying ∼50% of patients into the low‐risk group for whom an immediate subsequent surgical procedure could be avoided or postponed. Together, the assay we designed helps reduce unnecessary invasive monitoring and avoid repeated transurethral resection of BLCA (Re‐TURBT), not only alleviating the financial burden and the discomfort of patients but also mitigating the risk associated with unnecessary surgery. Hence, the accurate classification of cancer‐derived DNAm events not only helped elucidate molecular mechanisms underlying oncogenesis but also facilitated in development more sensitive and specific in vitro diagnostic assay highly relevant for clinical management of BLCA.

There are some limitations to this study. First, pathology assessment on resection samples may be limited by tumour heterogeneity, thus leading to the inconsistencies between UCAS and BLCAS prediction in the pathological evaluation. Because of tissue limitations, this study has performed complementary molecular classification on paired urine and tissue samples on only a few samples. Future clinical evaluations should include RNA‐ and immunohistochemistry (IHC)‐based molecular classification on resected tissue. Second, because of the scarce number of cases, the prediction ability of the urine DNAm signature for other pathological types of bladder tumours (neuroendocrine carcinoma, adenocarcinoma, sarcoma and others) as well as other types of urologic cancers were not analysed. Whether this assay could be applied to a wider range of urological cancers merits future investigation. Third, a 50% progression‐free survival (PFS) was not satisfied during our observation period for recurrence or metastasis. A longer follow‐up is needed to further substantiate the performance of the urine DNAm signature in monitoring the recurrence and metastasis of BLCA. Fourth, because only urines from healthy volunteers or patients subjected to provisional diagnostic or treatment via TURBT were included in the study, we have only a few PUMLMP and benign bladder disease patients in the final cohort. Such limitation is due to the enrolment criteria set before the clinical study. We expect future clinical trials to include a more general patient population, including microhaematuria, to test the general testing performance of the EUCAS assay. Finally, a throughout validation of oncogenic T2DMR should be carried out in the future.

In conclusion, we characterized DNAm identifiers of BLCA to enable accurate detection and classification of BLCA in urine. Our results not only could have practice‐changing implications for non‐invasive diagnosis and surveillance of BLCA but also implied widespread extended application of similar methods to other cancer types.

## CONFLICT OF INTEREST

All authors declare that they have no conflict of interest or financial conflicts to disclose.

## Supporting information

Supporting Information

Supporting Information
