# Supplementary material for: Non‐invasive diagnosis and surveillance of bladder cancer with driver and passenger DNA methylation in a prospective cohort study
Source: Clin Transl Med. 2022 Aug 15;12(8):e1008. doi: 10.1002/ctm2.1008 (PMC9377153; doi:10.1002/ctm2.1008)
Supplement: Supplementary file 1 — Supporting Information [file CTM2-12-e1008-s001.pdf]

Supplementary Figures

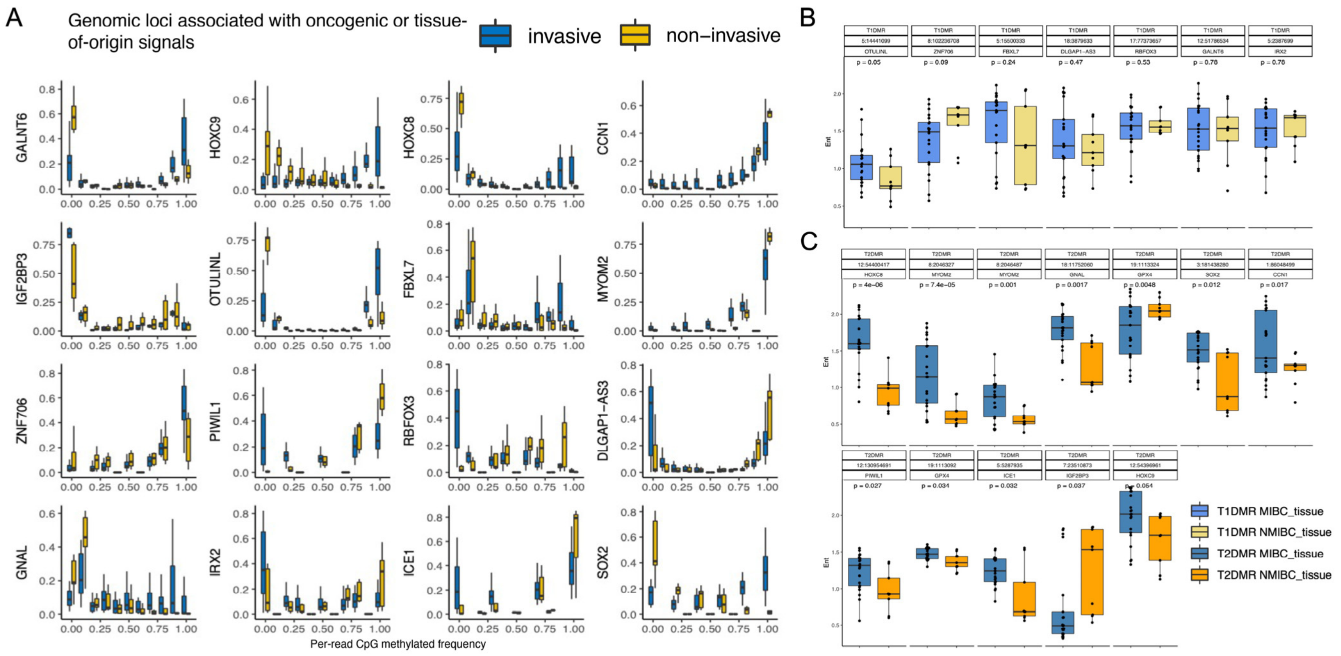

D

$$Ent_i = -1 * \sum_{j=0}^{n} p_{ij} * \log(p_{ij})$$

Where Ent_i is the entropy of i-th DMR
and p_{ij} is the haplotype frequency for j-th haplotype in i-th DMR
and n is the total number of haplotypes in i-th DMR

Supplementary Figure S1. Entropy of DNA methylation haplotypes distinguished Type I and II DMRs. (A) DMR-wise haplotype prevalence distribution in MIBC and NMIBC tissues. DMR-wise entropy of Type I **(B)** and Type II **(C)** DMR were calculated via formula **(D)** for each sample. Statistical significance was tested with t-test with FDR p-value adjustment. DMRs with sufficiently different entropy (P<0.1) between MIBC and NMIBC tissues are Type 2 DMR (T2DMR), whereas DMRs with indifferent entropy between MIBC and NMIBC tissues are Type I DMR (T1DMR).

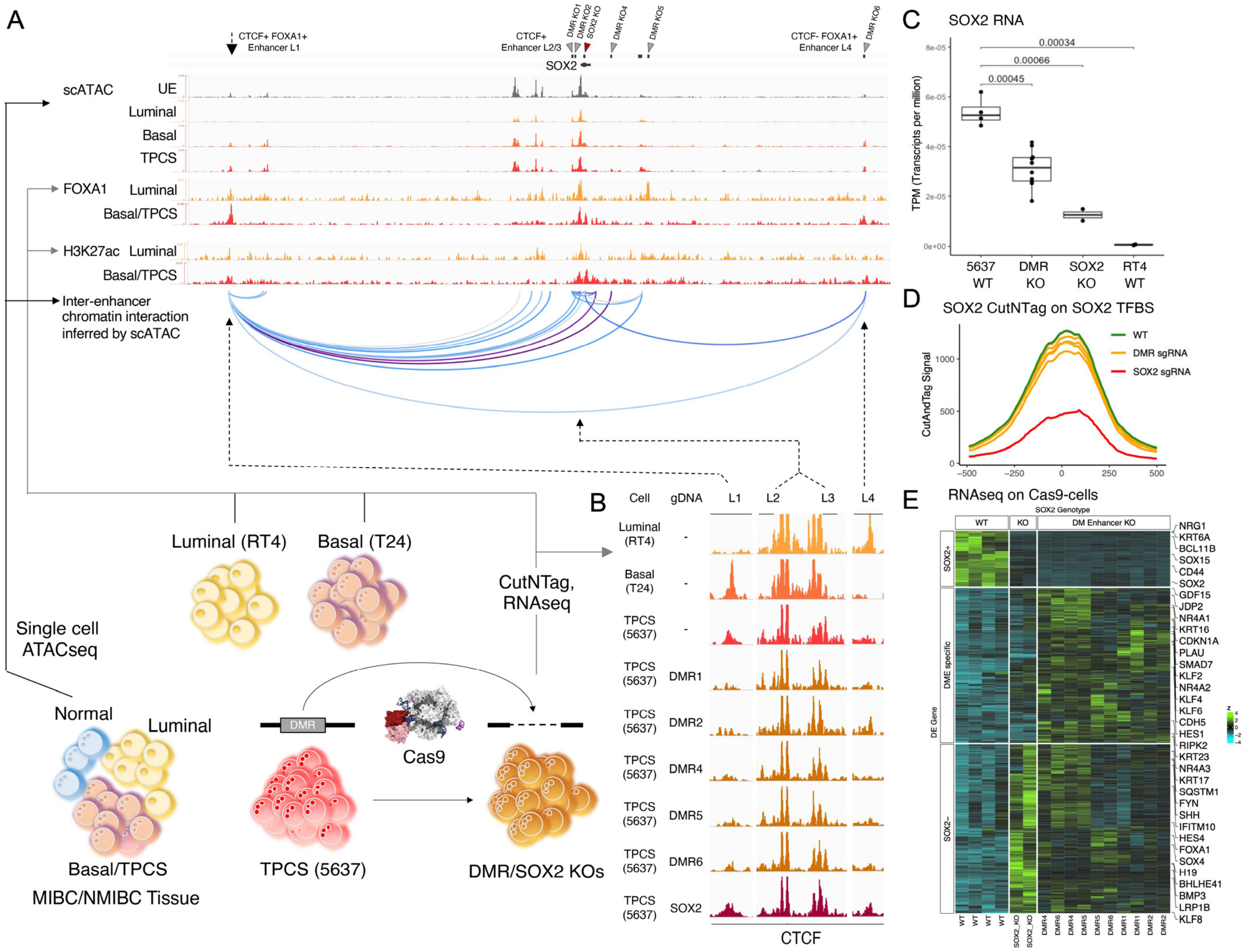

Supplementary Figure S2. Driver T2DMR interact with oncogene enhancer to control its function. (A) Aggregated single-cell ATAC-seq (top) track of normal urothelial cell (UE), luminal-type BLCA cell (Luminal), basal-type BLCA cell (Basal), and TM4SF1-positive hyperplastic cancer cell (TPCS), showing two basal/TPCS-specific FOXA1-bound (middle, FOXA1 CutNTag), H3K27ac-decorated (bottom, H3K27ac CutNTag) enhancers. gDNA position targeting SOX2 gene and its associated T2DMR are labelled on the top of panel. scATAC correlation indicates chromatin interaction between the 5' enhancer L1 and 3' enhancer L2 to T2DMR loci. **(B)** CTCF CutNTag on enhancers L1, L2, L3 and L4, with luminal (RT4), basal (T24), TPCS (5637), or TPCS with Cas9-KO of SOX2 or T2DMR. Whilst CTCF binding on L4 is luminal-specific and partially restored by either DMR2 KO or SOX2 KO, CTCF binding on L1 is basal/TPCS-specific, and is dependent on the presence of T2DMR. CTCF binding on L2 and L3 is dependent on DMR2. **(C)** Cas9-KO of SOX2 T2DMR significantly reduced SOX2 expression in TPCS. Both WT and KO cells contains the Cas9 transgene. **(D)** Cas9-KO of SOX2 T2DMR reduces SOX2 function in TPCS, as evident by reduced SOX2 CutNTag signal on its TFBS in T2DMR KO cells. **(E)** RNA expression changes in Cas9-KO of SOX2 T2DMR phenocopies SOX2 KO in TPCS.

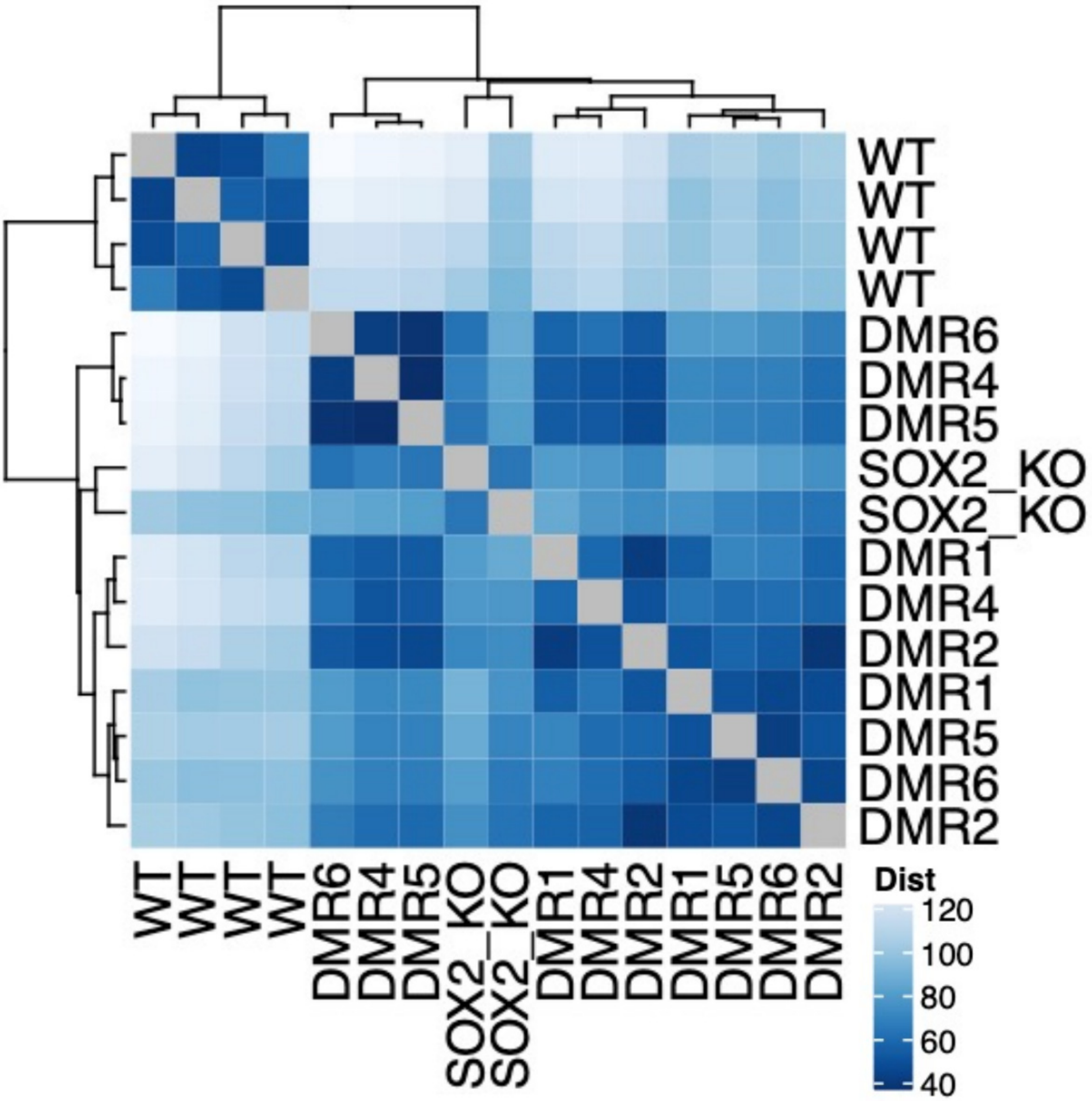

Supplementary Figure S3. RNA expression correlation between WT (Cas9^{+}, and Cas9^{-}), SOX2-gDNA KO, or DMR-gDNA KO TPCS (5637) cell lines, showing that KO of SOX2-associated T2DMR phenocopied SOX2 KO.

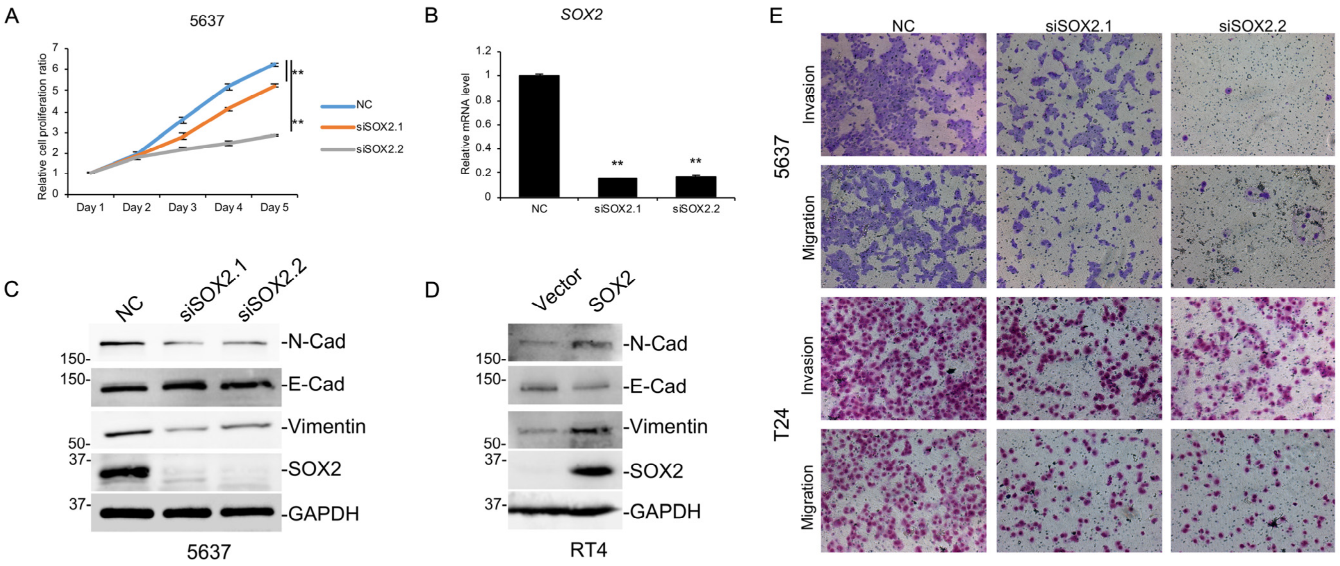

Supplementary Figure S4. SOX2 function is essential for MIBC aggressiveness. (A) 5637 growth curves with negative control siRNA (NC) or SOX2 siRNA (siSOX2.1/2). **(B)** SOX2 gene expression (quantified by qPCR) in NC, siSOX2.1 and siSOX2.2. **(C)** SOX2 protein expression in NC, siSOX2.1 and siSOX2.2. **(D)** SOX2 overexpression in luminal (RT4) cells results in elevated N-cadherin and vimentin expression, suggesting transformation towards aggressive phenotype. **(E)** Cell migration and invasion is severely affected by knockdown of SOX2.

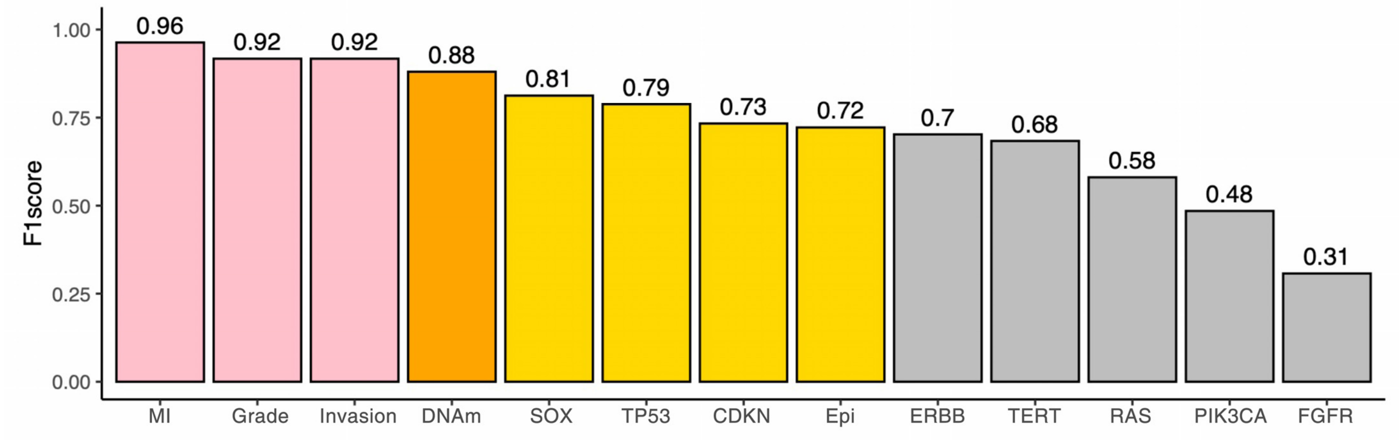

Supplementary Figure S5. F1 score of classification correctness for MIBC by each feature, showing that DNA methylation class outperforms mutations on any single gene in classifying NMIBC from MIBC.

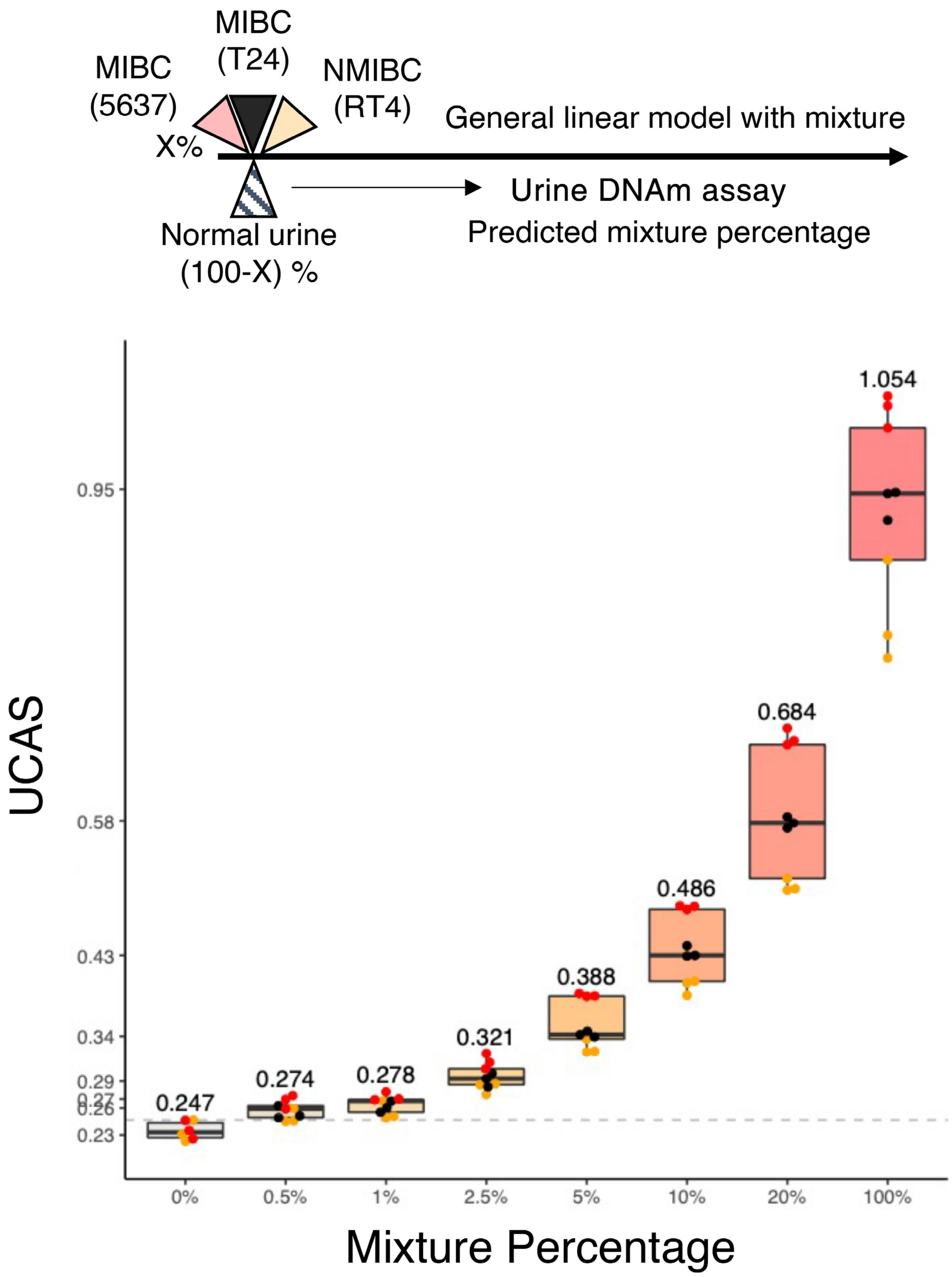

Supplementary Figure S6. Limits of detection for the multiplex amplicon NGS assay of BLCA-specific DMR DNA methylation. Genomic DNA from normal urine is mixed with predefined percentage of genomic DNA from blood cell (PBMC, mimicking hematuria), luminal-type BLCA (RT4) and basal-type BLCA (5637). General linear model was built with the tumor-associated DNA methylation haplotypes and used to predict mixture concentration. The assay shows excellent sensitivity and specificity for BLCA with LoD < 0.5% (the dashed line).

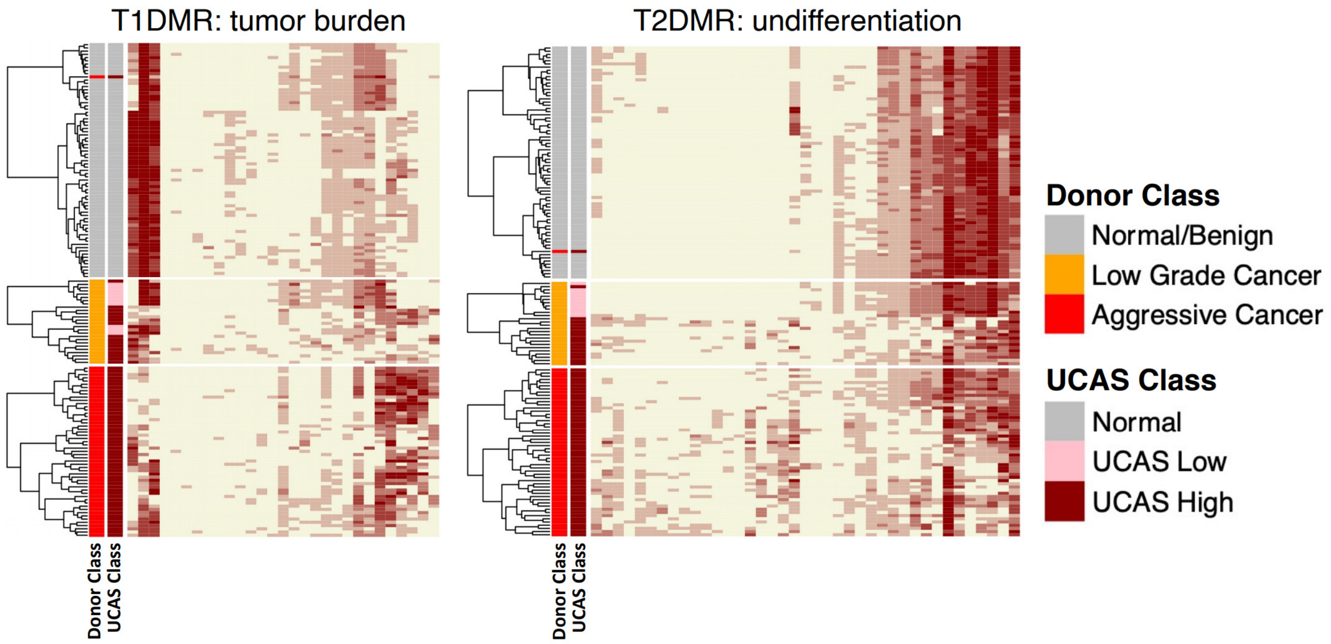

Supplementary Figure S7. Pre-surgery urine DNA cancer methylation score in different groups of samples suggesting false negative in urines from LG tumor donors are with low cancer-specific T2DMR haplotype prevalence, possibly from well-differentiated benign tumor.

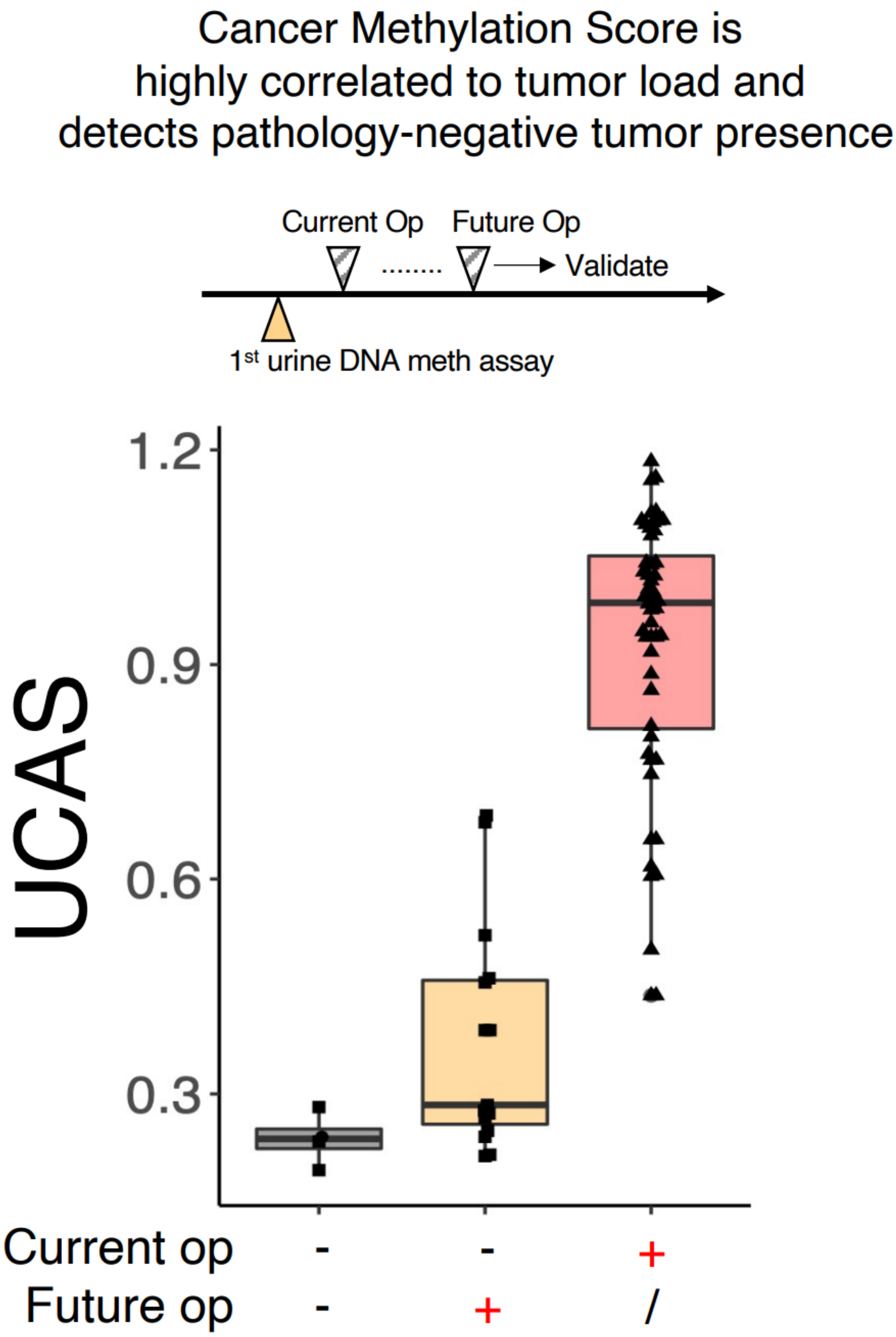

Supplementary Figure S8. Pre-surgery urine DNA methylation signal predicts tumor presence. UCAS scores from pre-surgery urines from patients who subsequently have two or more consecutive operations were shown. Patients with a positive pathology finding in the most immediate surgery (Current Op) have highest UCAS signal. UCAS signal from patients who showed a negative pathology finding in the immediate surgery but later turned out to be tumor-positive in subsequent surgery have intermediate UCAS signal. Patients without a positive pathology finding in all resected samples were UCAS negative.

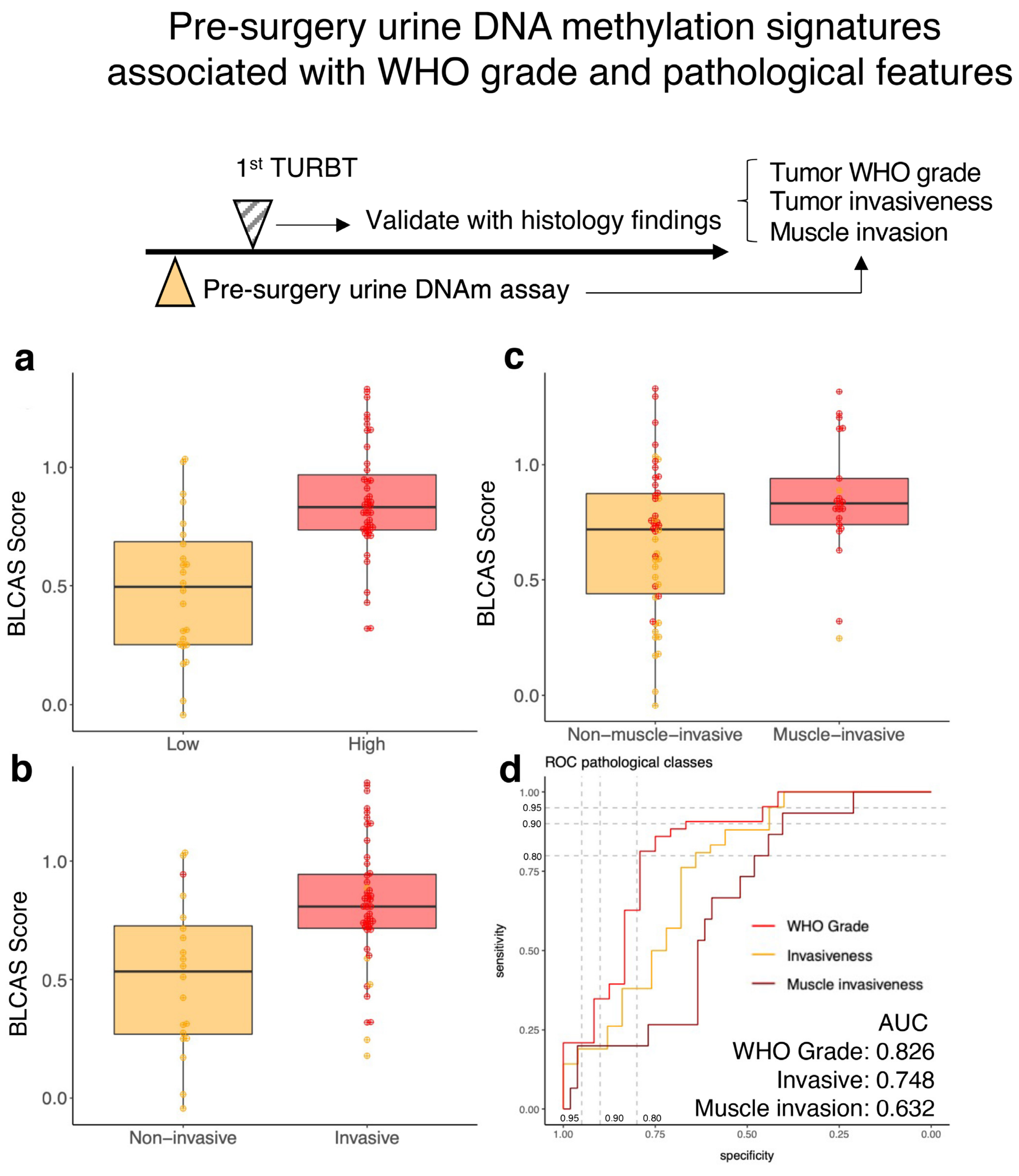

Supplementary Figure S9. Pre-surgery urine DNA methylation signal are associated with pathological features in resected tissue. Scores from a GLM model built with basal-specific DNA methylation haplotype load is able to distinguish pre-surgery urine samples from cancer patients of different WHO grade **(A)**, cell invasiveness **(B)**, muscle invasion **(C)**. Receiver-operating curve and AUC for each pathological feature predicted by DNA methylation signal is shown in **(D)**.

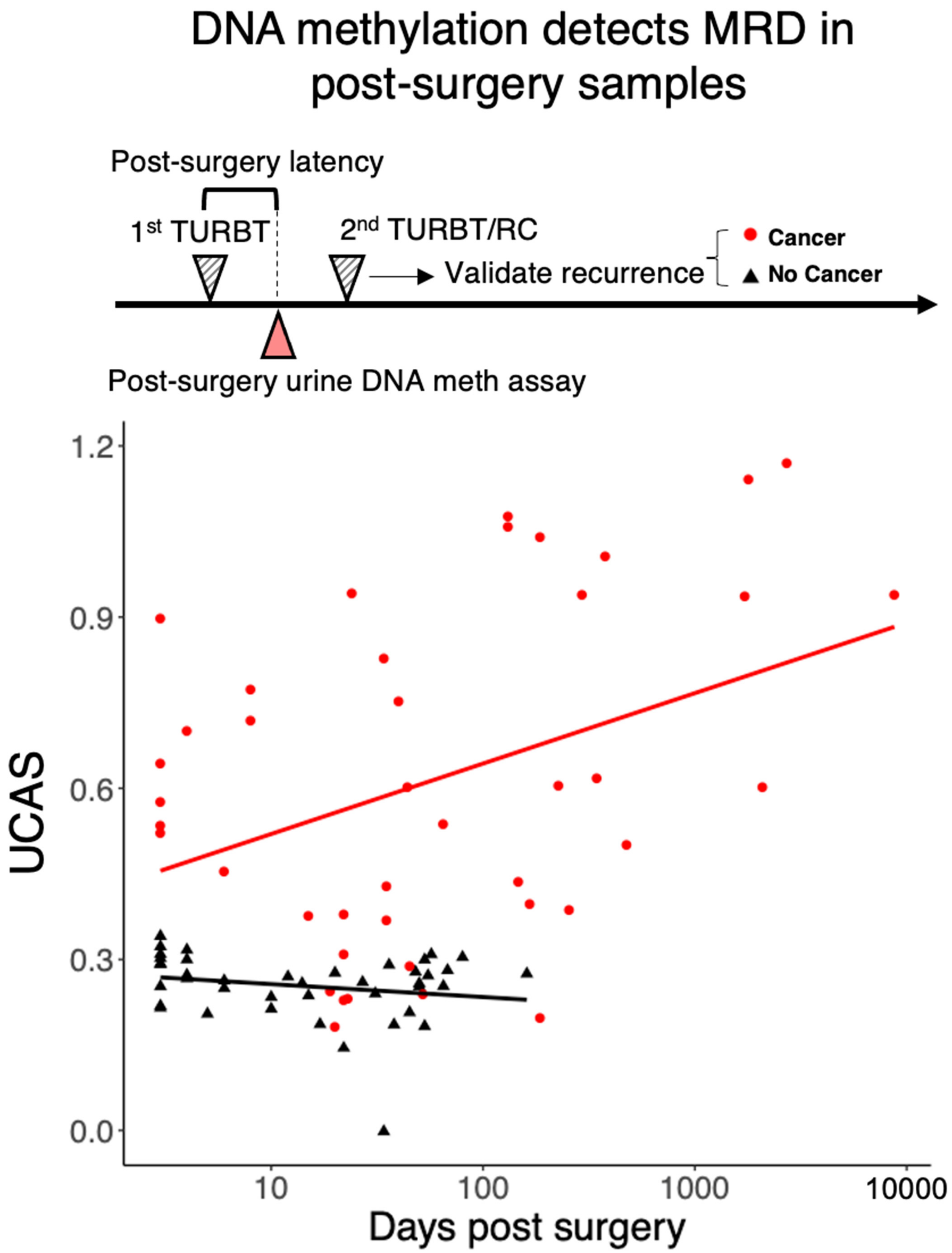

Supplementary Figure S10. Post-1st TURBT-surgery urine DNA methylation signal grows following a log-linear relationship to the post-surgery latency in patients with residual disease.
