# Supplementary material for: Non‐invasive diagnosis and surveillance of bladder cancer with driver and passenger DNA methylation in a prospective cohort study
Source: Clin Transl Med. 2022 Aug 15;12(8):e1008. doi: 10.1002/ctm2.1008 (PMC9377153; doi:10.1002/ctm2.1008)
Supplement: Supplementary file 2 — Supporting Information [file CTM2-12-e1008-s002.pdf]

Supplementary Table S1. Enrollment of the cohort.

	Patients	Healthy Donors
Total Screened	879	100
Unwilling to participate	426	16
Unable to provide consent	45	0
Patients who were discharged without treatment	17	0
Patient voluntarily left the study	27	0
Logistic issue	14	0
Insufficient quantity or quality	205	0
Enrolled Donor	145	84
Total collected tissue	33	0
Total collected urine	243	84
Insufficient urine cfDNA (<100ng)	51	5
Challenge-BLCA cohort: Donors	145	79
Samples	225	79
Validation cohort	192 (Urine)	67
Training cohort	33 (Tissue)	12

Supplementary Table S2. Aims of the study.

Experiment	Aim	Inclusion	Exclusion	n (screened)	n (excluded)	n (total)	Related to
Tissue DNA methylation profiling	Association of DNA methylation and mutation to pathology in BLCA tissue	1. Patients underwent surgery, and the surgical resection had enough tissue for research.<br>2. Biospecimens of surgical resection were cryopreserved.	1. No pathological diagnosis or pathological diagnosis was not BLCA (1: Neuroendocrine carcinoma)<br>2. <10% tumor content on pathological slide (3)	27	4	23	Figure 1; Figure 3; Figure S5
Pre-surgery Cancer Detection	Cancer detection in BLCA-history naive, pre-surgery urine	1.<br>a. Patients without prior BLCA history,<br>b. Clinical manifestations or imaging findings that suggested the possibility of BLCA.<br>c. Pre-surgery urine could be collected<br>d. Resection pathology report could be obtained<br>Or<br>2. Healthy donors without tumor history.	1. Patients without pathology reports (3)<br>2. Contaminated urine samples (2)	90+67 = 157	5	152	Figure 4; Figure S7
Cancer detection in pathology-negative patients	Predict HG BLCA presence in additional surgery with pre-surgery UCAS	1. Patients underwent a surgery and had pathological diagnosis.<br>2. Pre-surgery urine was collected.<br>3. Patients with confirmed pathological diagnosis of HG BLCA in first surgery, or despite no cancer in the first surgery, HG BLCA was pathologically confirmed in subsequent surgery or follow-up.	1. Patients without pathology reports (1)<br>2. Final pathological diagnosis was not BLCA (1: clear cell adenocarcinoma)<br>3. Final pathological diagnosis was LG BLCA (32)	109	34	75	Figure S8
Pre-surgery Pathology Prediction	Association between pre-surgery BLCAS and pathology traits in bladder urothelial carcinoma	1. Patients without prior BLCA history<br>2. Clinical manifestations or imaging findings that suggested the possibility of BLCA.<br>3. Pre-surgery urine could be collected<br>4. Resection pathology report could be obtained	1. BLCA with differentiation (1: adenocarcinoma, 1: plasmacytoid; 2: squamous; 1: neuroendocrine carcinoma;)<br>2. BLCA coexists with concurrent cancer (2: carcinoma of renal pelvis, 3: prostate cancer;<br>3. Benign tumor (2: PUNLMP)<br>4. High grade and low grade tumor coexist (1)<br>5. Contaminated urine samples (2)<br>6. No cancer was present in the pathological slide (5)<br>7. Patients without pathology reports (3)	90	23	67	Figure S9
Pre-surgery Prognosis Prediction	Predicts survival and metastasis with pre-surgery urine UCAS and BLCAS	1. Patients with confirmed pathological diagnosis of BLCA<br>2. Pre-surgery urine was collected.<br>3. Patients with complete postoperative follow-up data.	1. Clear-cell carcinoma (1)<br>2. Incomplete follow-up (81)	152	82	70	Figure 5
Residual Cancer Detection	Use paired pre-first-surgery and post-first-surgery urine to predict residual cancer	1. Patients with confirmed pathological diagnosis of BLCA and received at least two consecutive surgery within 3 months.<br>2. Pathological reports of two consecutive surgery were available.<br>3. Pathologically diagnosed as high grade BLCA in first surgery.<br>4. Paired pre-first-surgery and post-first-surgery urine was collected.	1. Not diagnosed as high grade BLCA (3)	16	3	13	Figure 6
Progression Prediction	Detect residual cancer and predict disease progression with post-surgery urine	1. Patients with confirmed pathological diagnosis of BLCA in a prior surgery<br>2. Post-surgery urine was collected.<br>3. Patients followed-up within 180 days of urine collection<br>4. Confirmatory negative of disease progression was determined by resection as well as clinical follow-up<br>5. Or: confirmatory positive of disease progression was determined by either resection or imaging	1. Patients with unclear evidence of recurrence or metastasis (1)<br>2. Unable to determine urine collection date (4)<br>3. Not assessed within 180 days of urine collection (29)	95	34	61	Figure 6

Supplementary Table S3. Training cohort.

Tissue donors in the training cohort	MIBC (n=14)	NMIBC (n=9)	Tumor Overall (n=23)	Normal Urine (n=12)
**Sex**				
F	2 (14.3%)	1 (11.1%)	3 (13.0%)	7 (58.3%)
M	12 (85.7%)	8 (88.9%)	20 (87.0%)	5 (41.7%)
**Age**				
Mean (SD)	61.9 (12.2)	53.4 (8.11)	58.6 (11.4)	60.6 (7.3)
Median [Min, Max]	60.0 [41.0, 84.0]	54.0 [33.0, 60.0]	56.0 [33.0, 84.0]	60.0 [24.0, 86.0]
**Grade**				
Low	0 (0%)	6 (66.7%)	6 (26.1%)	NA
Mixed	0 (0%)	1 (11.1%)	1 (4.3%)	NA
High	14 (100%)	2 (22.2%)	16 (69.6%)	NA
**Invasive**				
Non-invasive	0 (0%)	7 (77.8%)	7 (30.4%)	NA
Invasive	14 (100%)	2 (22.2%)	16 (69.6%)	NA
**Muscle invasion**				
No	1 (7.1%)	9 (100%)	10 (43.5%)	NA
Yes	13 (92.9%)	0 (0%)	13 (56.5%)	NA
**BLCA history**				
No	11 (78.6%)	6 (66.7%)	17 (73.9%)	12 (100%)
Yes	3 (21.4%)	3 (33.3%)	6 (26.1%)	0 (0%)
**TNM**				
TaN0Mx	0 (0%)	1 (11.1%)	1 (4.3%)	NA
TaNxMx	0 (0%)	6 (66.7%)	6 (26.1%)	NA
T1N0Mx	0 (0%)	2 (22.2%)	2 (8.7%)	NA
T2N0Mx	5 (35.7%)	0 (0%)	5 (21.7%)	NA
T2bN1Mx	1 (7.1%)	0 (0%)	1 (4.3%)	NA
T3N0Mx	2 (14.3%)	0 (0%)	2 (8.7%)	NA
T3aN0Mx	2 (14.3%)	0 (0%)	2 (8.7%)	NA
T3N2Mx	1 (7.1%)	0 (0%)	1 (4.3%)	NA
T4N0Mx	1 (7.1%)	0 (0%)	1 (4.3%)	NA
T4aN1Mx	1 (7.1%)	0 (0%)	1 (4.3%)	NA
T4aN2Mx	1 (7.1%)	0 (0%)	1 (4.3%)	NA

Supplementary Table S4. Validation cohort.

Urine samples with confirmed pathology in the validation cohort	Disease (n=192)	Healthy (n=67)
**Sex**		
Female	32 (16.7%)	41 (61.2%)
Male	160 (83.3%)	26 (38.8%)
**Age**		
Mean (SD)	67.1 (12.0)	62.7 (8.9)
Median [Min, Max]	68.0 [31.0, 93.0]	63.4 [23.0, 94.0]
**Cancer history**		
No	148 (77.0%)	67 (100%)
Prostate Cancer	1 (0.5%)	0 (0%)
Bladder Cancer	43 (22.4%)	0 (0%)
**Diagnosis (highest grade in history)**		
Bladder Inflammation	6 (3.1%)	0 (0%)
Inverted papilloma	1 (0.5%)	0 (0%)
PUNLMP	7 (3.6%)	0 (0%)
Bladder urothelial carcinoma (BLCA)	157 (81.8%)	0 (0%)
BLCA with adenocarcinoma differentiation	1 (0.5%)	0 (0%)
BLCA with squarmous differentiation	4 (2.1%)	0 (0%)
Plasmacytoid BLCA	1 (0.5%)	0 (0%)
Small cell cancer of the bladder	1 (0.5%)	0 (0%)
Clear cell adenocarcinoma of the bladder	2 (1.0%)	0 (0%)
Neuroendocrine carcinoma of the bladder	1 (0.5%)	0 (0%)
BLCA and carcinoma of renal pelvis	2 (1.0%)	0 (0%)
BLCA and prostate cancer	5 (2.6%)	0 (0%)
BLCA with squarmous differentiation and prostate cancer	1 (0.5%)	0 (0%)
Prostate cancer	1 (0.5%)	0 (0%)
Unknown	2 (1.0%)	0 (0%)
Healthy	0 (0%)	67 (100%)
**TNM**		
Benign tumor	1 (0.5%)	0 (0%)
TaN0Mx	1 (0.5%)	0 (0%)
TaNxMx	42 (21.9%)	0 (0%)
TaNxMx or T1NxMx	1 (0.5%)	0 (0%)
T1N0Mx	14 (7.3%)	0 (0%)
T1NxMx	25 (13.0%)	0 (0%)
T1NXMx	2 (1.0%)	0 (0%)
T2N0Mx	7 (3.6%)	0 (0%)
T2NxMx	10 (5.2%)	0 (0%)
T2aN0Mx	2 (1.0%)	0 (0%)
T2bN0Mx	1 (0.5%)	0 (0%)
T3N0Mx	1 (0.5%)	0 (0%)
T3N2Mx	4 (2.1%)	0 (0%)
T3aN0Mx	1 (0.5%)	0 (0%)
T3aN2Mx	1 (0.5%)	0 (0%)
T4aN0Mx	3 (1.6%)	0 (0%)
T4aN1Mx	1 (0.5%)	0 (0%)
T4aN2M1a	2 (1.0%)	0 (0%)
T4aN3Mx	1 (0.5%)	0 (0%)
T4bN2Mx	1 (0.5%)	0 (0%)
Nonapplicable	64 (33.3%)	67 (100%)
Unknown	7 (3.6%)	0 (0%)
**Tumor WHO Grade (where applicable)**		
Benign tumor	1 (0.5%)	0 (0%)
High	75 (39.1%)	0 (0%)
Low	48 (25.0%)	0 (0%)
Nonapplicable	63 (34.4%)	67 (100%)
Unknown	2 (1.0%)	0 (0%)
**Tumor invasiveness**		
Benign tumor	1 (0.5%)	0 (0%)
Invasive	76 (39.6%)	0 (0%)
Non-invasive	41 (21.4%)	0 (0%)
Nonapplicable	64 (33.3%)	67 (100%)
Unknown	10 (5.2%)	0 (0%)
**Muscle invasion**		
No	83 (43.2%)	0 (0%)
Yes	35 (18.2%)	0 (0%)
Nonapplicable	63 (34.4%)	67 (100%)
Unknown	10 (5.2%)	0 (0%)

Supplementary Table S5. siRNA used in the study.

Name	Sequence
siSOX2.1	5'-CCCUGCAGUACAACUCCAU-3'
siSOX2.2	5'-GGACAUGAUCAGCAUGUAU-3'

Supplementary Table S6. Antibodies used in the study.

Antibody	Manufacturer	Product code
H3K27ac	Abcam	ab4729
FOXA1	Abcam	ab170933
CTCF	Abcam	ab188408
SOX2	Abcam	ab92494
E-cadherin	CST	3195
N-cadherin	CST	13116
Vimentin	CST	5741
GAPDH	Santa Cruz	sc-365062
Goat-anti-mouse IgG	Sangon	D111024
Goat-anti-rabbit IgG	Sangon	D111018

Supplementary Table S7. sgDNA used in the study.

target_chromosome_GRCh38	target_region_start_GRCh38	target_region_end_GRCh38	target_name	sgDNA_1	sgDNA_2
chr3	181709493	181709993	DMR1	5'-ACATACAACCTCGAGGTAAG-3'	5'-AGTTTGACAGTAACAGGCTA-3'
chr3	181710227	181710727	DMR2	5'-TTATGGGAAGAAGGTTAGTA-3'	5'-GGCTGGGGCTGCGCCGCAAA-3'
chr3	181712474	181712974	SOX2 CDS	5'-GGCCCGCAGCAAACTTCGGG-3'	5'-GGTGGGCGAGCCGTTCATGT-3'
chr3	181719186	181719686	DMR4	5'-AAGTCTAAGATTCTTGTCTG-3'	5'-CCCGTGTCACGAGGTCAACA-3'
chr3	181728465	181728965	DMR5	5'-TACGTTAACTCCCACTGAGT-3'	5'-TTAATGACGCTATTACCATC-3'
chr3	181782231	181782731	DMR6	5'-TACATGACAGCAATTGCCTC-3'	5'-ATTCACACAGGGACTGTTAA-3'

All positions are in GRCh38

Supplementary Table S8. PCR primers.

Primer name	Forward Primer Seq	Reverse Primer Seq	Target region (GRCh37)
BLCBSP_V1-4	TTTCCCTACACGACGCTCTTCCGATCTNNNNNNNgtTGGATGTTTGAGTGTGAA	TTAATGCAACGATCGTCGAAATTCGCNNNNNNNCTTaaAAAATaTCTCCCCATCT	1:86048429-86048651
BLCBSP_V1-9	TTTCCCTACACGACGCTCTTCCGATCTNNNNNNNgGAGTGGTGGGGGATG	TTAATGCAACGATCGTCGAAATTCGCNNNNNNNCTCATTTATCCTaaAaCCTTTTC	7:23510805-23511072
BLCBSP_V1-13	TTTCCCTACACGACGCTCTTCCGATCTNNNNNNNGGtAGGGtTGGAATGAGAA	TTAATGCAACGATCGTCGAAATTCGCNNNNNNNCTCTaTTaCTAAaaCCCCAAAA	12:51786446-51786713
BLCBSP_V1-14	TTTCCCTACACGACGCTCTTCCGATCTNNNNNNNGTGAGGGtTGGGGAttATG	TTAATGCAACGATCGTCGAAATTCGCNNNNNNNCAaaaCTaTTCTACCATAACAAAC	12:54396863-54397136
BLCBSP_V1-15	TTTCCCTACACGACGCTCTTCCGATCTNNNNNNNTGAGGGATttttAAAAGGGG	TTAATGCAACGATCGTCGAAATTCGCNNNNNNNACCCTaCCAaAaAaCTATTCTT	12:54400340-54400617
BLCBSP_V2-7	TTTCCCTACACGACGCTCTTCCGATCTNNNNNNNGGtTtAGTTTTtAGAtAtTAGAAG	TTAATGCAACGATCGTCGAAATTCGCNNNNNNNAaaCTaaCCCTaTaaCCCCA	5:2387659-2387867
BLCBSP_V2-8	TTTCCCTACACGACGCTCTTCCGATCTNNNNNNNtTTTAtATAAAAttTTTGTGGATGG	TTAATGCAACGATCGTCGAAATTCGCNNNNNNNACCTaaATaCTTTaAaaCTaACCT	5:5287879-5288147
BLCBSP_V2-10	TTTCCCTACACGACGCTCTTCCGATCTNNNNNNNgTtTGGGTATTtttAtTTGTGAAA	TTAATGCAACGATCGTCGAAATTCGCNNNNNNNCAaaaACAaaCACACAaACTaaC	5:14441021-14441266
BLCBSP_V2-11	TTTCCCTACACGACGCTCTTCCGATCTNNNNNNNgGGGAttYgtAAtAAGTGG	TTAATGCAACGATCGTCGAAATTCGCNNNNNNNcRaaCAaaCCCCTaaaCTC	5:15500260-15500461
BLCBSP_V2-16	TTTCCCTACACGACGCTCTTCCGATCTNNNNNNNGTGTGGttTGAGTGttTGTT	TTAATGCAACGATCGTCGAAATTCGCNNNNNNNAaTCCTTCACTCAATCCCAC	8:2046233-2046598
BLCBSP_V2-17	TTTCCCTACACGACGCTCTTCCGATCTNNNNNNNGGAAATtAtTTTGGGGATTGAG	TTAATGCAACGATCGTCGAAATTCGCNNNNNNNTaaACCAAATATTTCAaaAAaaACC	8:2046233-2046598
BLCBSP_V2-18	TTTCCCTACACGACGCTCTTCCGATCTNNNNNNNAGGAAGGGGtTttAGttAAAG	TTAATGCAACGATCGTCGAAATTCGCNNNNNNNCCTaaAaCAAaaATCAaaATaTCC	8:102236659-102236863
BLCBSP_V2-19	TTTCCCTACACGACGCTCTTCCGATCTNNNNNNNGGtAGGAGAAGGttAtTtATG	TTAATGCAACGATCGTCGAAATTCGCNNNNNNNCTTTTTAAaaTATaACACCCATCC	12:130954602-130954836
BLCBSP_V2-21	TTTCCCTACACGACGCTCTTCCGATCTNNNNNNNgAGGAAtTttttTTTGGTAGGA	TTAATGCAACGATCGTCGAAATTCGCNNNNNNNCCCTaATTACAATaCTaaCACT	17:77373510-77373785
BLCBSP_V2-22	TTTCCCTACACGACGCTCTTCCGATCTNNNNNNNgAtTTGGTGAAGAGtTTtTGG	TTAATGCAACGATCGTCGAAATTCGCNNNNNNNTaaCTATCACACCCTaCAaTAC	18:3879388-3879760
BLCBSP_V2-24	TTTCCCTACACGACGCTCTTCCGATCTNNNNNNNGtTGAGYgAGGtTTGGAG	TTAATGCAACGATCGTCGAAATTCGCNNNNNNNAAATCTaCTaaATaAATACAaCACA	18:11751967-11752237
BLCBSP_V2-33	TTTCCCTACACGACGCTCTTCCGATCTNNNNNNNTGtTGGGAAAGGGAGGG	TTAATGCAACGATCGTCGAAATTCGCNNNNNNNCCCAaCTTCACTTTTTCACT	19:1113012-1113477
BLCBSP_V2-35	TTTCCCTACACGACGCTCTTCCGATCTNNNNNNNTGGGGtTTAGAGGGATGG	TTAATGCAACGATCGTCGAAATTCGCNNNNNNNTCTaaTaaCCCCTaCTaaTCAC	19:1113012-1113477
MN-DMR4-1	TTTCCCTACACGACGCTCTTCCGATCTNNNNNNNGTTAGGGTTTGTAGGGTTT	TTAATGCAACGATCGTCGAAATTCGCNNNNNNNTAATAAAACTAAACTACACTAACCTC	3:181438100-181438500

For qRT-PCR

Primer name	Forward Primer Seq	Reverse Primer Seq
β-Actin	GATCCACATCTGCTGGAAG	CAGCACAATGAAGATCAAGA
SOX2	GGAAACTTTTGTCGGAGAC	TATTTATAATCCGGGTGCTC

All positions are in GRCh37

Supplementary Table S9. Softwares.

R packages	base 3.6.2
AnnotationDbi	1.48.0
ArchR	0.9.5
Biobase	2.46.0
BiocGenerics	0.32.0
BiocParallel	1.20.1
Biostrings	2.54.0
BSgenome	1.54.0
BSgenome.Hsapiens.1000genomes.hs37d5	0.99.1
ChIPseeker	1.22.1
ComplexHeatmap	2.2.0
data.table	1.12.8
DESeq2	1.26.0
DelayedArray	0.12.3
dplyr	1.0.6
GenomeInfoDb	1.22.1
GenomicFeatures	1.38.2
GenomicRanges	1.38.0
ggbeeswarm	0.6.0
ggbiplot	0.55
ggplot2	3.3.5
ggpubr	0.4.0
ggrepel	0.8.2
ggsci	2.9
gtsummary	1.5.0
IRanges	2.20.2
jsonlite	1.7.1
knitr	1.3
lsei	1.2-0
magrittr	1.5
Matrix	1.2-18
matrixStats	0.57.0
MLmetrics	1.1.1
openxlsx	4.1.5
org.Hs.eg.db	3.10.0
patchwork	1.0.1
pheatmap	1.0.12
plotly	4.9.2.1
plyr	1.8.6
pROC	1.16.2
Rsamtools	2.2.3

readr	1.3.1
rhdf5	2.30.1
rtracklayer	1.46.0
S4Vectors	0.24.4
scales	1.1.1
scoringutils	0.1.7.2
stringr	1.4.0
SummarizedExperiment	1.16.1
survival	3.2-3
survminer	0.4.8
survMisc	0.5.5
table1	1.2
tidyr	1.1.3
TxDb.Hsapiens.UCSC.hg19.knownGene	3.2.2
XVector	0.26.0

Python packages	base 3.8.3
Pysam	0.16.0.1
Numpy	1.21.0

Linux packages	
bwa (Sentieon)	0.7.17-r1188
Sentieon package	sentieon-genomics-201911
PileOMeth (MethylKit)	0.1.13-3-gca82747
HISAT	2.1.0
STAR	2.5.3a
SAMBAMBA	0.5.4
MACS2	2.2.7.1
Bowtie2	2.2.3
Genrich	0.6
VEP	90.1
SnpSift	4.2
Pisces	5.2.9.122
CNVKit	0.7.11
SAMBLASTER	0.1.22
BEDTOOLS	v2.25.0
HTSLIB	1.2.1

Mac packages	
IGV	2.6.3

Supplementary Table S10. Cancer-specific methylation score (cancer methylation score) of individual samples.

pcr_lib_name	final_diagnosis	TNM	grade	UCAS
0726AMP-U-9	blc_ca	TaNxMx	low	0.111322921
0824AMP-U-4	blc_ca	TaNxMx	low	0.168881955
0917AMP-U-38	healthy	no_cancer	no_cancer	0.172133149
0919AMP-U-5	healthy	no_cancer	no_cancer	0.179674419
0820AMP-U-1	blc_ca	TaNxMx	low	0.189946658
1019AMP-U-22	healthy	no_cancer	no_cancer	0.190705997
0820AMP-U-16	bladder_inflammation	no_cancer	no_cancer	0.193638049
0824AMP-U-3	blc_ca	TaNxMx	low	0.199611999
0917AMP-U-35	healthy	no_cancer	no_cancer	0.218531621
0924AMP-U-19	blc_ca	TaNxMx	low	0.219557298
1019AMP-U-25	healthy	no_cancer	no_cancer	0.224115109
1019AMP-U-23	healthy	no_cancer	no_cancer	0.224979424
0917AMP-U-43	healthy	no_cancer	no_cancer	0.232685716
1019AMP-U-18	healthy	no_cancer	no_cancer	0.233484625
0824AMP-U-6	bladder_inflammation	no_cancer	no_cancer	0.23365714
0917AMP-U-34	healthy	no_cancer	no_cancer	0.240584353
0909AMP-U-19	Inverted Papilloma	benign tumor	benign tumor	0.240842191
0917AMP-U-51	healthy	no_cancer	no_cancer	0.243841771
0909AMP-U-30	healthy	no_cancer	no_cancer	0.244720423
0917AMP-U-46	healthy	no_cancer	no_cancer	0.255648964
1019AMP-U-38	healthy	no_cancer	no_cancer	0.256884064
0917AMP-U-36	healthy	no_cancer	no_cancer	0.260816256
1019AMP-U-43	healthy	no_cancer	no_cancer	0.263153589
0917AMP-U-31	healthy	no_cancer	no_cancer	0.26469413
1019AMP-U-32	healthy	no_cancer	no_cancer	0.266067963
1019AMP-U-33	healthy	no_cancer	no_cancer	0.267284243
1019AMP-U-30	healthy	no_cancer	no_cancer	0.268122924
0917AMP-U-10	blc_ca	TaNxMx	low	0.268871265
1019AMP-U-34	healthy	no_cancer	no_cancer	0.269077649
0917AMP-U-42	healthy	no_cancer	no_cancer	0.270094996
0917AMP-U-45	healthy	no_cancer	no_cancer	0.273329843
0917AMP-U-12	blc_ca	TaNxMx	low	0.273421175
0909AMP-U-20	blc_ca	TaNxMx	low	0.280828488
0917AMP-U-18	bladder_inflammation	no_cancer	no_cancer	0.281612295
0917AMP-U-56	healthy	no_cancer	no_cancer	0.283474274
1019AMP-U-29	healthy	no_cancer	no_cancer	0.287062962
0919AMP-U-2	healthy	no_cancer	no_cancer	0.289087316
1019AMP-U-40	healthy	no_cancer	no_cancer	0.290145883
1019AMP-U-21	healthy	no_cancer	no_cancer	0.290849388
0820AMP-U-22	blc_ca	T1NxMx	low	0.29505181
1019AMP-U-37	healthy	no_cancer	no_cancer	0.295651659
1019AMP-U-28	healthy	no_cancer	no_cancer	0.296718948
0917AMP-U-48	healthy	no_cancer	no_cancer	0.301508369
1019AMP-U-26	healthy	no_cancer	no_cancer	0.302188075
0917AMP-U-50	healthy	no_cancer	no_cancer	0.302733803
1019AMP-U-35	healthy	no_cancer	no_cancer	0.30625186
0919AMP-U-3	healthy	no_cancer	no_cancer	0.308686036
0917AMP-U-47	healthy	no_cancer	no_cancer	0.308905496
0917AMP-U-39	healthy	no_cancer	no_cancer	0.310599723
1019AMP-U-36	healthy	no_cancer	no_cancer	0.314409893
0909AMP-U-27	healthy	no_cancer	no_cancer	0.315846423
0909AMP-U-28	healthy	no_cancer	no_cancer	0.316529763
0917AMP-U-30	healthy	no_cancer	no_cancer	0.316650932
1019AMP-U-31	healthy	no_cancer	no_cancer	0.316783576
0917AMP-U-53	healthy	no_cancer	no_cancer	0.319260093
1019AMP-U-17	healthy	no_cancer	no_cancer	0.319636738
0917AMP-U-41	healthy	no_cancer	no_cancer	0.319836206
0917AMP-U-44	healthy	no_cancer	no_cancer	0.321678088
0917AMP-U-49	healthy	no_cancer	no_cancer	0.332677098
0917AMP-U-37	healthy	no_cancer	no_cancer	0.332877316
0917AMP-U-40	healthy	no_cancer	no_cancer	0.334575707
0917AMP-U-52	healthy	no_cancer	no_cancer	0.336299261
0917AMP-U-32	healthy	no_cancer	no_cancer	0.336498587
1019AMP-U-39	healthy	no_cancer	no_cancer	0.341696856
1019AMP-U-20	healthy	no_cancer	no_cancer	0.345436672
0909AMP-U-31	healthy	no_cancer	no_cancer	0.349227952
1019AMP-U-27	healthy	no_cancer	no_cancer	0.350235859
1019AMP-U-42	healthy	no_cancer	no_cancer	0.351684214

pcr_lib_name	final_diagnosis	TNM	grade	UCAS
0909AMP-U-29	healthy	no_cancer	no_cancer	0.352423247
0917AMP-U-55	healthy	no_cancer	no_cancer	0.353106492
0917AMP-U-54	healthy	no_cancer	no_cancer	0.353409874
1019AMP-U-41	healthy	no_cancer	no_cancer	0.35373649
0909AMP-U-23	blc_ca	T1NxMx	low	0.356480607
1019AMP-U-24	healthy	no_cancer	no_cancer	0.360566303
0919AMP-U-1	healthy	no_cancer	no_cancer	0.367030756
0917AMP-U-33	healthy	no_cancer	no_cancer	0.370671046
0919AMP-U-4	healthy	no_cancer	no_cancer	0.375239464
1019AMP-U-19	healthy	no_cancer	no_cancer	0.376671737
0924AMP-U-11	blc_ca	no_cancer	no_cancer	0.389044419
1019AMP-U-44	healthy	no_cancer	no_cancer	0.393743997
0917AMP-U-57	healthy	no_cancer	no_cancer	0.401441528
0909AMP-U-26	healthy	no_cancer	no_cancer	0.407848116
0917AMP-U-26	cca_ca clear cell adenocarcinoma	T2NxMx	cca	0.437116807
0924AMP-U-10	blc_ca_plasmacytoid	T3aN0Mx	high	0.43748228
0917AMP-U-6	blc_ca_PUNLMP	TaNxMx	low	0.456285059
0917AMP-U-17	blc_ca	T1NxMx	high	0.501325205
0723AMP-U-2	blc_ca	T4aN1Mx	high	0.537564493
0820AMP-U-23	blc_ca	T1NxMx	high	0.604054344
0820AMP-U-27	blc_ca	T2NxMx	high	0.605779859
0820AMP-U-25	blc_ca_squamous_diff	T4aN0Mx	high	0.617306183
0818AMP-U-9	blc_ca	T1NxMx	high	0.655285501
0824AMP-U-5	blc_ca	T4bN2Mx	high	0.655372549
0917AMP-U-1	blc_ca	T1N0Mx	high	0.702109555
0917AMP-U-16	blc_ca	TaNxMx	low	0.74052813
0917AMP-U-29	blc_ca	T1N0Mx	high	0.746294526
0818AMP-U-4	blc_ca	T2NxMx	high	0.766246234
0917AMP-U-21	blc_ca	TaNxMx	high	0.766549484
0917AMP-U-3	blc_ca	TaNxMx	low	0.771034211
0909AMP-U-25	blc_ca	T1NxMx	high	0.774580455
0917AMP-U-9	blc_ca	T4aN2M1a	high	0.798899902
0824AMP-U-9	blc_ca	TaNxMx	low	0.849415449
0820AMP-U-11	blc_ca	T2NxMx	high	0.863786573
0824AMP-U-8	blc_ca	T2NxMx	high	0.88652685
0818AMP-U-11	blc_ca and carcinoma of renal pelvis	T1NxMx	high	0.898934465
0723AMP-U-6	blc_ca	T3N2Mx	high	0.917817358
0820AMP-U-24	blc_ca	T1N0Mx	high	0.938671912
0909AMP-U-22	blc_ca	T2NxMx	high	0.938810419
0820AMP-U-12	blc_ca	T1NxMx	high	0.940896099
0723AMP-U-5	blc_ca	T1N0Mx	high	0.946470047
0820AMP-U-21	net_ca neuroendocrine carcioma	T2NxMx	high	0.958713868
0924AMP-U-21	blc_ca	TaNxMx	low	0.958951367
0917AMP-U-8	blc_ca	TaNxMx	low	0.97289104
0723AMP-U-14	blc_ca	T2NoMx	high	0.976920704
0723AMP-U-16	blc_ca	T2N0Mx	high	0.978604038
0909AMP-U-24	blc_ca_adeno_diff	T2aN0Mx	high	0.985162322
0820AMP-U-19	blc_ca	T0.5NxMx	high	0.987578697
0726AMP-U-3	blc_ca	T1N0Mx	high	0.989045779
0726AMP-U-10	blc_ca	T1NXMx	high	0.994691489
0824AMP-U-15	blc_ca and prostate_ca	T1NxMx	high	0.995389603
0924AMP-U-16	blc_ca	T1N0Mx	high	1.002134259
0824AMP-U-14	blc_ca	unknown	low	1.003420255
0818AMP-U-12	blc_ca and carcinoma of renal pelvis	T1NxMx	high	1.006214556
0917AMP-U-22	blc_ca	T2NxMx	high	1.017528597
0917AMP-U-7	blc_ca	TaNxMx	low	1.022703475
0824AMP-U-16	blc_ca	T4aN0Mx	high	1.02443161
0917AMP-U-15	blc_ca	T2N0Mx	high	1.02527835
0924AMP-U-17	blc_ca	T1NxMx	high	1.029814375
0917AMP-U-5	blc_ca	TaNxMx	low	1.039149268
0726AMP-U-2	blc_ca	T1NXMx	high	1.039617309
0917AMP-U-4	blc_ca_PUNLMP	TaNxMx	low	1.041589255
0824AMP-U-10	blc_ca and prostate_ca	T1NxMx	high	1.042225302
0723AMP-U-19	blc_ca	T1NxMx	high	1.042492884
0917AMP-U-13	blc_ca	TaNxMx	low	1.0625362
0924AMP-U-15	blc_ca	TaNxMx	low	1.068969977
0909AMP-U-10	blc_ca	T1NxMx	low	1.070514905
0917AMP-U-28	blc_ca	TaNxMx	low	1.077601298
0820AMP-U-14	blc_ca_squamous_diff	T3N2Mx	high	1.080386104

pcr_lib_name	final_diagnosis	TNM	grade	UCAS
0818AMP-U-21	blc_ca	T1N0Mx	high	1.087379304
0818AMP-U-16	blc_ca and prostate_ca	T1N0Mx	high	1.0910169
0924AMP-U-18	blc_ca	T1NxMx	high	1.09620463
0917AMP-U-11	blc_ca	TaNxMx	low	1.097908935
0924AMP-U-14	blc_ca	T1NxMx	high	1.09871145
0924AMP-U-20	blc_ca	unknown	high	1.10120424
0820AMP-U-8	blc_ca	unknown	high	1.101716308
0818AMP-U-5	blc_ca	T1NxMx	high	1.10208939
0820AMP-U-17	blc_ca	T1NxMx	high	1.112060215
0924AMP-U-31	blc_ca	T1NxMx	high	1.114212721
0909AMP-U-21	blc_ca	TaNxMx	low	1.142934661
0917AMP-U-19	blc_ca	T1NxMx	high	1.15759988
0824AMP-U-1	blc_ca	T2bN0Mx	high	1.161356685
0820AMP-U-15	blc_ca	T1NxMx	high	1.171507126
0820AMP-U-13	blc_ca	T1N0Mx	high	1.183928843

Supplementary Table S11. Number of patients.

Figure	Prediction Target	High Group Definition	Low Group Definition	High Group N	Low Group N
Figure 5A-1	Recurrence	UCAS Positive	UCAS Negative	57	13
Figure 5A-2	Recurrence	High Grade Tumor	Low Grade Tumor	43	27
Figure 5A-3	Recurrence	Cell Invasive	Cell non-invasive	42	28
Figure 5A-4	Recurrence	Muscle layer invasion	No muscle layer invasion	16	54
Figure 5B-1	Metastasis	BLCAS Positive	BLCAS Negative	49	21
Figure 5B-2	Metastasis	High Grade Tumor	Low Grade Tumor	43	27
Figure 5B-3	Metastasis	Cell Invasive	Cell non-invasive	42	28
Figure 5B-4	Metastasis	Muscle layer invasion	No muscle layer invasion	16	54
